# Perspective of potential patients on the hospital volume-outcome relationship and the minimum volume threshold for total knee arthroplasty: a qualitative focus group and interview study

**DOI:** 10.1186/s12913-021-06641-8

**Published:** 2021-07-02

**Authors:** Charlotte M. Kugler, Karina K. De Santis, Tanja Rombey, Kaethe Goossen, Jessica Breuing, Nadja Könsgen, Tim Mathes, Simone Hess, René Burchard, Dawid Pieper

**Affiliations:** 1 Institute for Research in Operative Medicine, Witten/Herdecke University, Ostmerheimer Str. 200, 51109 Cologne, Germany; 2 Leibniz Institute for Prevention Research and Epidemiology- BIPS, Department: Prevention and Evaluation, Achterstr. 30, 28359 Bremen, Germany; 3 Department of Trauma Surgery and Orthopaedics, Lahn-Dill-Kliniken, Rotebergstr. 2, 35683 Dillenburg, Germany; 4 Department of Health, Witten/Herdecke University, Alfred-Herrhausen-Straße 50, 58448 Witten, Germany; 5 School of Medicine, Univerity of Marburg, Baldingerstraße, 35032 Marburg, Germany

**Keywords:** Total knee arthroplasty, Volume-outcome relationship, Minimum volume threshold, Hospital choice, Hospital quality data, Public involvement

## Abstract

**Background:**

Total knee arthroplasty (TKA) is performed to treat end-stage knee osteoarthritis. In Germany, a minimum volume threshold of 50 TKAs/hospital/year was implemented to ensure outcome quality. This study, embedded within a systematic review, aimed to investigate the perspectives of potential TKA patients on the hospital volume-outcome relationship for TKA (higher volumes associated with better outcomes).

**Methods:**

A convenience sample of adults with knee problems and heterogeneous demographic characteristics participated in the study. Qualitative data were collected during a focus group prior to the systematic review (*n* = 5) and during telephone interviews, in which preliminary results of the systematic review were discussed (*n* = 16). The data were synthesised using content analysis.

**Results:**

All participants (*n* = 21) believed that a hospital volume-outcome relationship exists for TKA while recognising that patient behaviour or the surgeon could also influence outcomes. All participants would be willing to travel longer for better outcomes. Most interviewees would choose a hospital for TKA depending on reputation, recommendations, and service quality. However, some would also choose a hospital based on the results of the systematic review that showed slightly lower mortality/revision rates at higher-volume hospitals. Half of the interviewees supported raising the minimum volume threshold even if this were to increase travel time to receive TKA.

**Conclusions:**

Potential patients believe that a hospital volume-outcome relationship exists for TKA. Hospital preference is based mainly on subjective factors, although some potential patients would consider scientific evidence when making their choice. Policy makers and physicians should consider the patient perspectives when deciding on minimum volume thresholds or recommending hospitals for TKA, respectively.

**Supplementary Information:**

The online version contains supplementary material available at 10.1186/s12913-021-06641-8.

## Background

### Prevalence of total knee arthroscopy

Knee osteoarthritis is a common chronic condition that affected 263 million people globally in 2017 [1]. The years lived with disability due to knee osteoarthritis increased by 31% between 2007 and 2017 [1]. Total knee arthroplasty (TKA) is a successful therapy for end-stage knee osteoarthritis, resulting in pain relief and functional improvement [2]. In Germany, 190,427 primary TKAs and 25,097 revision TKAs were performed in 2018, and primary TKA ranked 14th among the inpatient surgeries performed most frequently [3].

### Hospital volume-outcome relationship

Previous research has shown that a relationship exists between hospital volume and the health outcomes of certain surgical procedures [4–7], but the existence of this relationship for TKA has not yet been demonstrated [8]. Hospital volume is a proxy for various characteristics of the hospital, such as processes and infrastructure [9]. There are two hypotheses to explain the association between higher hospital volumes and better health outcomes. According to the first hypothesis (“practice makes perfect”), higher hospital volumes result in greater experience and therefore better skills of the entire team [4, 5, 10] and in greater standardisation of processes [9]. As a consequence, higher volumes lead to better health outcomes. According to the second hypothesis (“selective referral”), patients are referred to hospitals known for good health outcomes [4]. Therefore, good health outcomes lead to higher volumes in such hospitals [4]. The reasons why high-volume hospitals achieve better outcomes are still not fully understood [9].

### Minimum volume thresholds and their consequences

In Germany, a minimum volume of 50 TKA procedures per hospital site per year needs to be performed to receive reimbursement for TKA surgeries from statutory health insurers [11]. However, the minimum volume threshold had low effects on healthcare due to low compliance by hospitals in the past [12, 13]. From the patient perspective, a minimum volume threshold has the potential to increase safety by reducing mortality and complications. However, this increase in safety can occur at the expense of the availability of healthcare services, thereby increasing the travel burden for patients and their carers. In particular, smaller hospitals in rural areas are threatened regarding their financial viability and their ability to recruit and retain surgeons [14]. A study conducted prior to the implementation of the minimum volume threshold found that 13 and 31% of TKA patients would have to be redistributed to other hospitals if the threshold were 50 and 100 TKAs per hospital, respectively [15]. Although minimum volume thresholds could affect local healthcare for a large number of patients, the patient perspective has been neglected in the debate on this issue [16, 17].

### Hospital choice of TKA patients

Many European countries introduced policy reforms to strengthen the role of patients in selecting their healthcare providers [18–21]. Free choice is one aspect of patient empowerment and thereby a goal in its own right [19, 20]. Indeed, the opportunity to choose a hospital for TKA is associated with patient satisfaction with surgery [22], and TKA patients also prefer to select a surgeon themselves [23]. The patient as a consumer is assumed to choose only those providers that offer the best care based on publicly available comparative information [20, 24]. However, the decision-making process of patients is complex and influenced by many factors [25]. In general, patients consider a variety of structural, process and quality characteristics of providers [25]. Knee or hip arthroplasty patients rely on their own experience, recommendations by others (friends, family, and primary care providers), or information they find on the internet or in magazines [26, 27]. When candidates for hip or knee replacement surgery decide on a hospital, they consider outcome quality (infection rates, complication rates) [26], postoperative pain management [26], and other factors such as kindness of the staff [26], surgeon reputation [23] and waiting time [23]. Travel time is also important [23], but TKA patients were willing to travel a longer time to lower their surgical risk in a discrete choice experiment [17]. However, views of patients regarding the hospital volume-outcome relationship in TKAs remain unclear.

### Objective of the current study

The objectives of this qualitative study were threefold: 1) to investigate the perspectives of potential patients on the existence of a hospital volume-outcome relationship, 2) to assess their consequences for their choice of hospital for TKA and 3) to explore the implications of a potential hospital volume-outcome relationship on the minimum volume threshold for TKA in Germany. The study is embedded within our systematic review of the hospital volume-outcome relationship for TKA (PROSPERO protocol CRD42019131) [8], in which we aim to investigate and quantify the relationship between hospital volume and patient-relevant outcomes following TKA. Public involvement in our systematic review is particularly important [28] because a hospital volume-outcome relationship and the minimum volume threshold for TKA could affect a large number of patients. The opinions of those ultimately affected by the minimum volume threshold regulation should be considered by regulators [29]. With the current study, we aim to supplement our quantitative findings of the systematic review with the perspectives of potential patients as consumers in the healthcare system.

## Methods

The current study adheres to the Consolidated Criteria for Reporting Qualitative Research recommendations (COREQ, Additional file 1) [30]. The characteristics of the research team are described in Additional file 2.

The study was conducted in two phases: Phase 1, prior to our systematic review, and Phase 2, once the preliminary results of our systematic review were available. During Phase 2, we incorporated the scientific findings from our systematic review in personalised hospital risk sheets to discuss the preferences for choosing healthcare providers for TKA with potential patients.

### Participants

The study was approved by the Research Ethics Committee at Witten/Herdecke University, Germany (approval number 54/2019). The participants were recruited using convenience and snowball sampling strategies. We planned three focus groups with eight participants each because the optimal size of focus groups is between five and ten [31]. The eligibility criteria for both phases of the study were as follows: 1) age of at least 18 years, and 2) knee problems, including pain and reduced functionality, either already treated with TKA or potentially requiring TKA in the future. We intended to include primarily participants without TKA because patients already treated with TKA could potentially be biased due to their experience [26]. All participants gave their written informed consent to participate in the study and received compensation of 12.50 €.

#### Phase 1: prior to the systematic review

Phase 1 of the study included one focus group. The participants were recruited using information leaflets distributed at the Department of Trauma and Orthopaedic Surgery, Cologne-Merheim Medical Centre (CMMC), University of Witten/Herdecke, Germany, and by word of mouth by researchers from our institute. The participants of the focus group did not know each other or the main moderator (JB) prior to the study.

#### Phase 2: after the systematic review

Since minimum volume thresholds can differentially affect people residing in rural compared to urban areas, we planned two focus groups, one involving participants from urban areas and one from rural areas, classified based on their home address. Due to contact restrictions during the SARS-CoV-2 pandemic in early 2020, we had to replace two focus groups with individual telephone interviews but maintained the planned number of participants (2 focus groups × 8 participants = 16 participants in total). The participants were recruited by word of mouth by researchers from our institute. Recruited participants recommended other potential participants from their social networks. The interviewees did not know the interviewer (TR) prior to the study. The interviews were conducted only once with each participant.

### Material

The focus group and interview guides were semistructured and selfdeveloped based on key questions defined in the protocol [8] (Table 1). The interview guide was pretested with one person to ensure that the questions were understandable.

**Table 1:** Focus group and interview guides

Focus group guide (discussion topics)	Interview guide (questions)
**1)** ^†^ We have just heard some reasons why you might need to have such surgery [TKA]. What do you conclude from this? What are the reasons for having TKA?	**1)** How many TKAs do you think a hospital performs on average annually?
**2) **How many TKAs do you think a hospital performs on average annually?	**2)** What number represents a low volume of TKAs for you?
**3)** What number represents a low volume of TKAs for you?	**3)** What number represents a high volume of TKAs for you?
**4)** What number represents a high volume of TKAs for you?	**4)** Please comment on the following statement: “The more TKAs a hospital performs, the better the outcome of the TKA”. • In your opinion, which factors influence the outcome of the TKA? • How do you explain the relationship between the number of TKAs performed and the outcomes?
**5) **Please comment on the following statement: “The more TKAs a hospital performs, the better the outcome of the TKA” • In your opinion, which factors influence the outcome of a TKA? • How do you explain the relationship between the number of TKAs performed and the outcomes?	**5)** Let us say you have a choice between two different hospitals. Hospital A is in your immediate vicinity, so you do not have to travel a long distance. Hospital B is much further away, which means you have a longer travel time. Hospital B has better outcomes of the surgery than Hospital A. Would you be willing to travel longer to hospital to obtain better outcomes of the surgery?
**6)** ^†^ What would you say if you were to have TKA now and you were informed about the volume of TKAs in the chosen hospital? With what number would you be satisfied, or would you feel comfortable with to say: ‘I will have my surgery there’. What is an acceptable number of surgeries a year for you to say: ‘Yes, they can do it, the result will be good; I will be in good hands’?	**6)** What is the maximum travel time you would accept to achieve a better outcomes?
**7) **How long on average do you drive to the nearest hospital from where you live?	**7)** What other factors (besides distance and case number) would influence your decision to choose a hospital?
**8)** How many hospitals do you have in your area?	**8)** [Preliminary results of a systematic review are presented to the participant via a previously sent ‘summary of results’ document (Fig. 8) and the explanation (Fig. 9) over the phone]. After explaining the results: Would you say that the number of TKAs/hospital/year has a major impact on the outcomes?
**9)** Let us say you have a choice between two different hospitals. Hospital A is in your immediate vicinity, so you do not have to travel a long distance. Hospital B is much further away, which means you have a longer travel time. Hospital B has better outcomes of the surgery than Hospital A. Would you be willing to travel longer to hospital for better outcomes of the surgery?	**9) **Based on our calculations, would you definitely rule out one or more of the 3 hospitals if you had planned to have a TKA?
**10)** What is the maximum travel time you would accept to obtain better outcomes?	**10)** Currently, there is a minimum volume threshold for TKAs in Germany. This means that a hospital has to do at least 50 TKAs/year to receive reimbursement for these surgeries. What would be your opinion if the minimum volume threshold were set to [a number above the number from the 1st hospital] per year and [1st hospital] would no longer be allowed to perform this surgery?

**Note.** We introduced the term TKA at the beginning of the focus group and the interviews but referred to the procedure using the more general term ‘knee joint surgery’ throughout the study to improve the understanding of the participants. ^†^ Added during the focus group. Underlined text = words accentuated during interview. TKA = total knee arthroplasty

### Study process

**Fig. 1:**
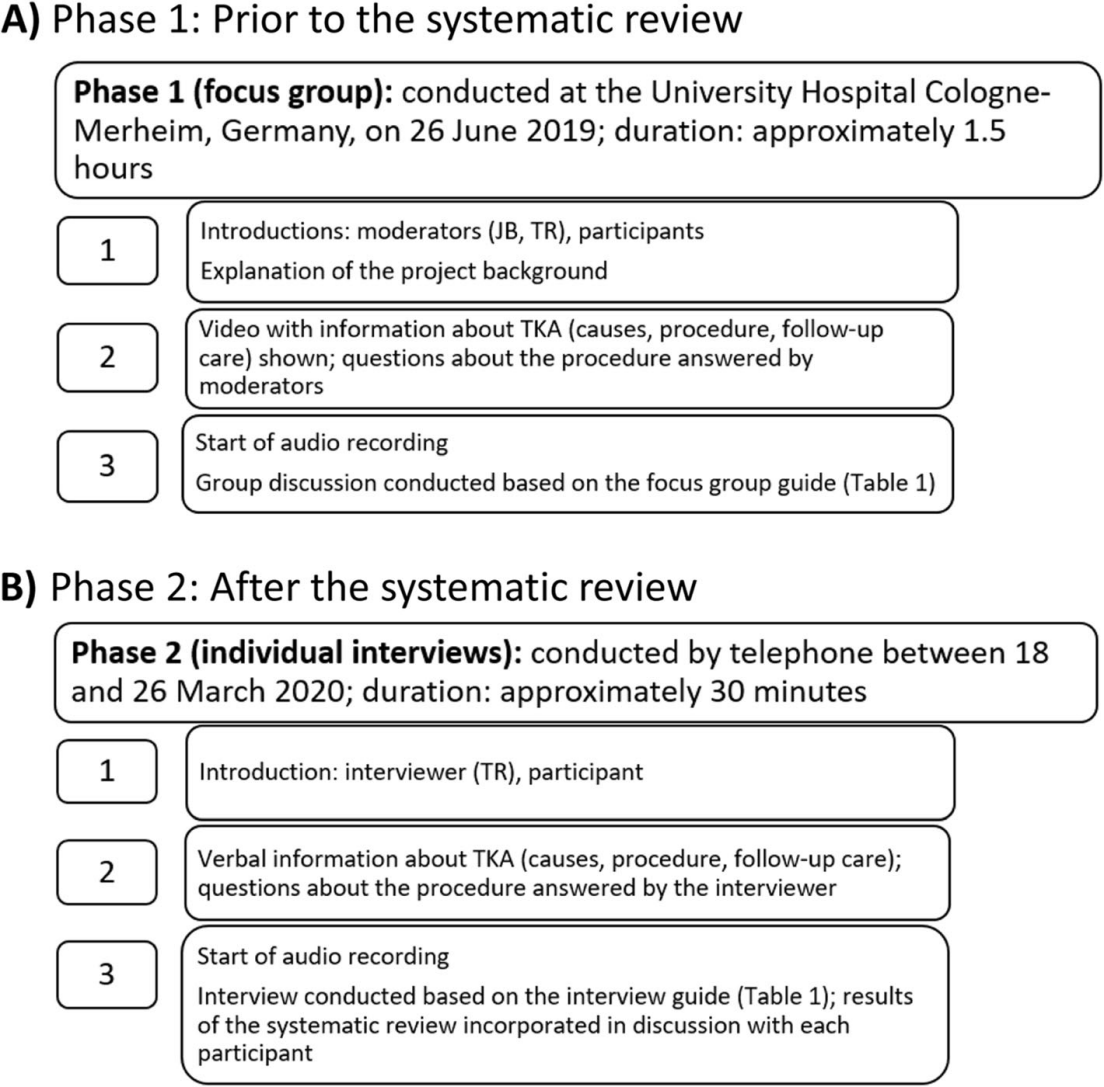
A. Study process of phase 1: prior to the systematic review. The video shown during the focus group can be accessed online [32]. B. Study process of phase 2: after the systematic review. Eligibility was established over the telephone before participants were invited to participate in the study. TKA = total knee arthroplasty

The process applied in the current study is illustrated in Fig. 1. After introduction of the moderators or interviewer, information about TKA was presented by video [32] or verbally. The focus group and the interviews were audio-recorded using an OLYMPUS Boundary Microphone ME33 (Shinjuku, Tokyo, Japan) and OLYMPUS Digital Voice Recorder WS-853 (Shinjuku, Tokyo, Japan). A student assistant also took notes during the focus group to supplement the audio recording but did not actively participate in discussion.

### Data processing and analysis

**Fig. 2:**
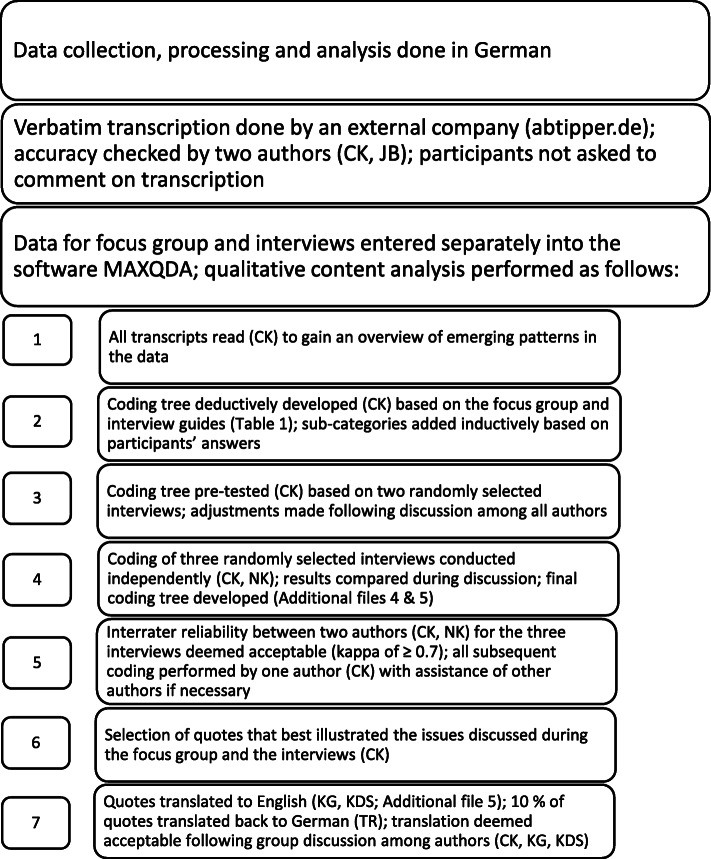
Data processing and analysis. Qualitative content analysis according to Mayring [33] is described in detail elsewhere. We used MAXQDA [34] for coding and computing kappa [35, 36]

All data were saved on a server at Witten/Herdecke University, Germany. The steps involved in data processing and analysis are illustrated in Fig. 2. Focus group and interviews were transcribed verbatim and synthesised using qualitative content analysis according to Mayring [33]. We used MAXQDA software [34] to code and analyse the data.

### Participant feedback

Once all data were analysed, we invited one participant from Phase 2 of the study who was suitable according to the interviewer (TR) for an additional feedback session in accordance with COREQ recommendations [30]. The aim of the session was to validate our interpretation of the answers of the participant and to ask for potential information sources that the participant would use to search for hospital quality data. Due to contact restrictions during the SARS-CoV-2 pandemic, the session was conducted by one researcher (CK) via video call, while a second researcher (KG) took notes.

## Results

### Demographic characteristics of all study participants

The demographic characteristics of all 21 participants are reported in Table 2. They were heterogeneous in terms of age, gender, level of education and prior experience with TKA. We involved participants from rural and urban areas.

**Table 2:** Demographic characteristics of participants

	Phase 1: Focus group	Phase 2: Interviews		
		rural residence	urban residence	total
Sample size (*n)*	5	7	9	16
**Gender**				
Female	4	2	4	6
Male	1	5	5	10
**Age (years)**				
< 60	2	6	3	9
≥ 60	3	1	6	7
**School graduation**				
None	0	0	0	0
Lower secondary school	1	1	1	2
Intermediate secondary school	1	0	3	3
Tertiary entrance qualification	3	6	5	11
**TKA**				
Previous TKA	2	0	2	2
No previous TKA	3	7	7	14

Note. There were *n* = 6 participants recruited for Phase 1 and *n* = 18 participants recruited for Phase 2 (one and two participants, respectively, were unable to attend the scheduled appointments). Most participants were from North Rhine-Westphalia, a relatively densely populated area with no proper rural areas compared to other regions in Germany [37]. The cutoff for ‘urban’ was more than 500 inhabitants per km^2^ [38]. Despite this cutoff, we reclassified one place of residence from urban to rural due to distance to a larger city (> 150,000 inhabitants) and low availability of infrastructure such as hospitals. TKA = total knee arthroplasty

The participants of Phase 1 (focus group) were mainly female and 56 years old on average (range: 28–73 years). The participants in Phase 2 (interviews) were mainly male, 64 years old on average (range: 25–76 years) and included mainly urban residents. All participants had at least lower secondary school certificates. The majority of participants had no previous TKA.

### Phase 1: prior to the systematic review (focus group)

According to our discussion guide (Table 1), three main topics were addressed during the focus group: hospital TKA volumes, hospital volume-outcome relationship and hospital preference (travel time vs. hospital quality) for TKA.

#### Hospital volumes

Participants had difficulties estimating hospital TKA volumes in Germany. They estimated that the average number of TKAs per hospital was between 150 and 1500 TKAs, and these values were thought to depend on the hospital size. Participants defined low hospital TKA volumes as 50 to 100 TKAs and high hospital volumes as 500 to 1800 TKAs. This definition was often based on their own calculations, as illustrated in the following quote:

*‘I was just thinking this through, with three doctors operating in the whole hospital. And they would do two surgeries a day each. And then, when I calculate 300 days, let's say...’ (B4)*

#### Hospital volume-outcome relationship

**Fig. 3:**
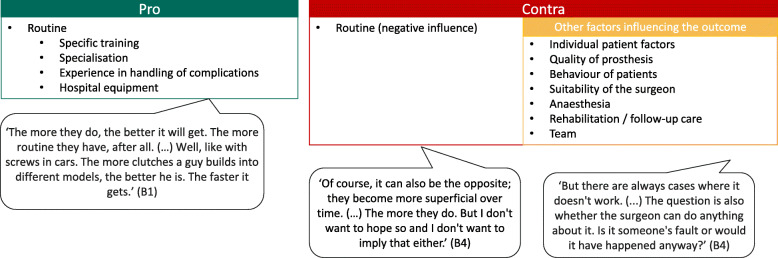
Phase 1 (focus group): Hospital volume-outcome relationship. Discussion topic 5: ‘Please comment on the following statement: “The more TKAs a hospital performs, the better the outcome of the TKA”. In your opinion, which factors influence the outcome of a TKA? How do you explain the relationship between the number of TKAs performed and the outcomes?’ (*n* = 5 participants). B = participant identification number; TKA = total knee arthroplasty

All participants acknowledged the existence of a hospital volume-outcome relationship for TKA (Fig. 3). They associated higher hospital TKA volumes with better TKA outcomes, mainly due to routine but thought that other factors such as individual patient factors could also influence the outcomes.

#### Hospital preference (travel time vs. hospital quality)

**Fig. 4:**
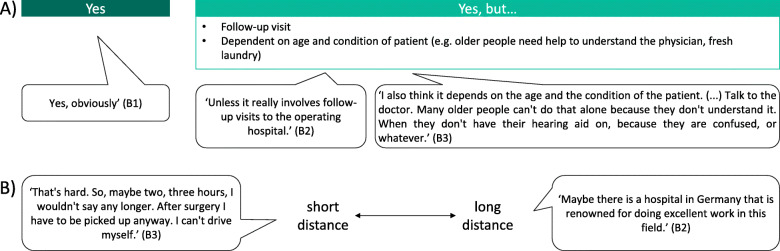
Phase 1 (focus group): Hospital preference (travel time vs. hospital quality). A) Discussion topic 9: ‘Let us say you have a choice between two different hospitals. Hospital A is in your immediate vicinity, so you do not have to travel long distances. Hospital B is much further away, which means you have a longer travel time. Hospital B has a better surgical outcome than Hospital A. Would you be willing to travel longer to the hospital for better surgical outcomes?’ B) Discussion topic 10: ‘What is the maximum travel time you would accept to obtain better outcomes?’ (*n* = 5 participants). B = participant identification number

When given a choice between two different hospitals, one in the immediate vicinity and one further away but with better outcomes of the surgery, all participants were willing to travel longer to obtain better health outcomes (Fig. 4). The acceptable maximum travel time to obtain better outcomes varied among the participants, from a few hours to Germany- or Europe-wide travel. However, they mentioned that the willingness of some patients to travel longer might be limited: for example, older people may depend on transport and help from relatives.

### Phase 2: after the systematic review (interviews)

The interviews were conducted once the preliminary results of our systematic review were available (see section 3.3.5). According to our interview guide (Table 1), five main topics were discussed during the interviews: hospital TKA volumes, a hospital volume-outcome relationship for TKA, hospital preference (travel time vs. hospital quality), factors affecting hospital choice, and the preliminary results of our systematic review.

#### Hospital volumes

Similar to the focus group, the interviewees also had difficulties estimating hospital TKA volumes in Germany. The average annual number of TKAs per hospital was estimated to be 30 to 2000 TKAs (median: 275), often based on their own calculations:

*‘If one works 200 days, that's as many in 1 day, then. Or it could even be two a day. Oh, I'd say 400, then.’ (B20)*

Participants defined low hospital TKA volumes as 0 to 100 TKAs (median of 75) and high hospital TKA volumes as 100 to 15,000 TKAs (median of 925).

#### Hospital volume-outcome relationship

**Fig. 5:**
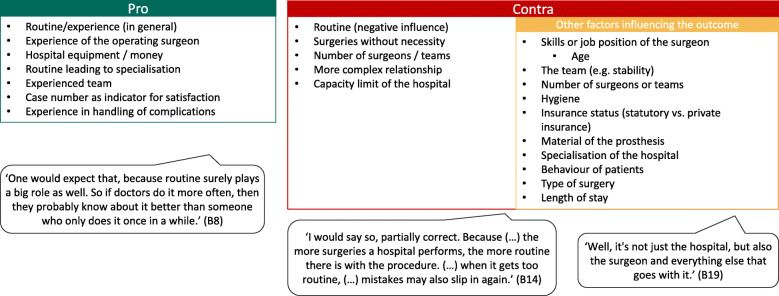
Phase 2 (interviews): Hospital volume-outcome relationship. Question 4: ‘Please comment on the following statement: “The more TKAs a hospital performs, the better the outcome of the TKA”. In your opinion, which factors influence the outcome of a TKA? How do you explain the relationship between the number of TKAs performed and the outcomes?’ (*n* = 16). B = participant identification number; *n* = number of participants; TKA = total knee arthroplasty

All interviewees acknowledged the existence of a hospital volume-outcome relationship for TKA (Fig. 5). The main reasons why higher TKA volumes were associated with better TKA outcomes were the increased routine, experience of the operating surgeons and teams, and facilities with superior equipment. However, some interviewees thought that higher hospital TKA volumes could also negatively influence TKA outcomes, for example, when routine leads to lower attention to detail or when surgeries are performed without necessity. Some also thought that other factors would influence the outcomes more strongly than hospital TKA volume.

#### Hospital preference (travel time vs. hospital quality)

**Fig. 6:**
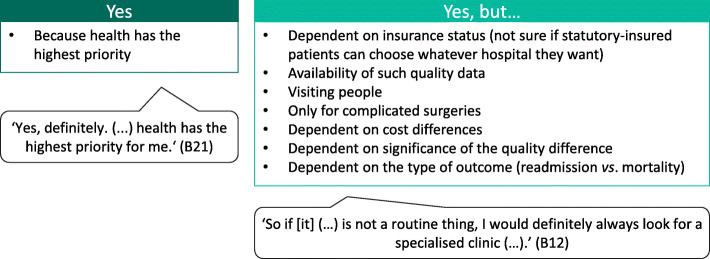
Phase 2 (interviews): Hospital preference (travel time vs. hospital quality). Question 5: ‘Let us say you have a choice between two different hospitals. Hospital A is in your immediate vicinity, so you do not have to travel long distances. Hospital B is much further away, which means you have a longer travel time. Hospital B has a better surgical outcome than Hospital A. Would you be willing to travel longer to a hospital for better surgical outcomes?‘(*n* = 15 and *n* = 1 with missing data). B = participant identification number; *n* = number of participants

When given a choice between two different hospitals, one in the immediate vicinity and one further away but with better outcomes of the surgery, all interviewees were willing to travel longer to obtain better health outcomes (Fig. 6). The acceptable maximum travel time to obtain better outcomes of the surgery varied from 35 min to approximately 6 hours (Germany-wide). However, some interviewees limited their willingness to travel longer to some preconditions, for example, a complex surgery, the availability of hospital quality data and the type of outcome that differed between hospitals.

We compared the opinions of potential patients according to their area of residence (urban vs. rural). The range of acceptable travel times did not vary between the groups, but on average, participants from rural areas were willing to travel longer.

#### Factors affecting hospital choice

**Fig. 7:**
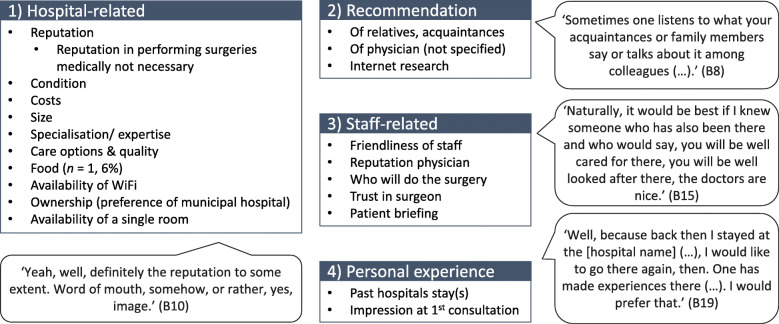
Phase 2 Factors affecting hospital choice. Question 7: ‘What other factors (besides distance and case number) would influence your decision to choose a hospital?‘(*n* = 15 and *n* = 1 with missing data). B = participant identification number; *n* = number of participants

In addition to hospital volume, travel time and quality, hospital choice for TKA also depended on subjective factors (Fig. 7). The most commonly mentioned topic was hospital-related factors, including reputation, condition of the hospital or its size. The second topic concerned recommendations by relatives, acquaintances, physicians or the internet. The third topic was staff-related factors, including friendliness and reputation of staff. The fourth topic was personal experience from past hospitalisations or first consultation.

#### Discussion of results of the systematic review

**Fig. 8:**
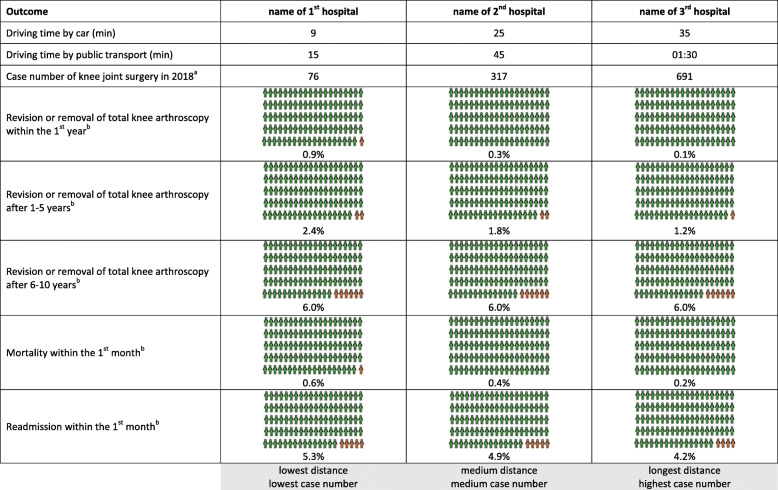
Personalised hospital risk sheet for discussion of results from our systematic review. The figure shows an example of a personalised hospital risk sheet. We presented the results of our systematic review in the context of real case numbers from three actual hospitals located in the residence area of each participant. The first hospital was always the nearest, which – by chance – was always the one with the smallest case number for TKAs, the second hospital was intermediate regarding both distance and case number for TKAs, while the third hospital was always the furthest one with the highest case number for TKAs. ^a^Real case numbers for TKAs per hospital. These values were only given to the interviewees during the interview to avoid influencing the first questions of the interview regarding the estimation of case numbers. ^b^Values computed based on the preliminary results of our systematic review. TKA = total knee arthroplasty

We discussed the preliminary results of our systematic review with the interviewees using personalised hospital risk sheets provided prior to the interviews (Fig. 8).

**Fig. 9:**
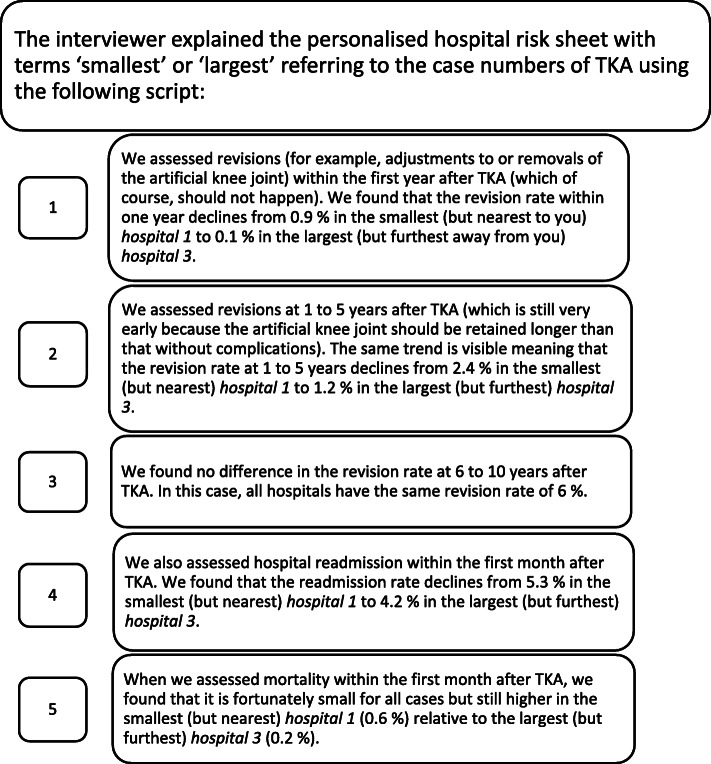
Explanation of the personalised hospital risk sheet. The explanation refers to the hospital risk sheet presented in Fig. 8. The script was read by the interviewer (TR). TKA = total knee arthroplasty

The preliminary dose-response meta-analysis in our systematic review produced odds ratios per ten additional TKAs per year for various harms outcomes. We combined these odds ratios and the absolute risk data from the included studies with real hospital TKA volumes in 2018 [39] to compute risk rates for five harms outcomes for three actual hospitals located in the residence area of each participant (Fig. 8). This procedure was applied to simulate a “real-life” decision based on hospitals the interviewees knew so that they were able to evaluate whether the personalised hospital risk sheets would influence their hospital choice. We presented frequencies and used pictograms for illustration [40] to let interviewees choose which form of presentation was best for them. The explanation of the personalised hospital risk sheets that were given to the interviewees is summarised in Fig. 9. The interviewer also informed the participant of the real hospital TKA volumes of the presented hospitals.

**Fig. 10:**
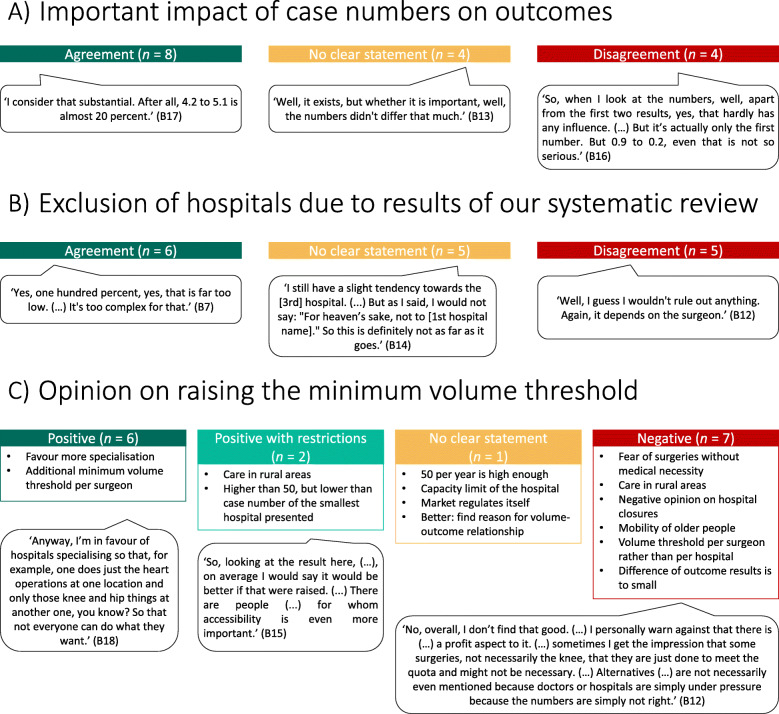
Phase 2 (interviews) Interpretation of results from our systematic review. After explaining the personalised hospital risk sheet: A) Question 8: Would you say that the number of TKAs/hospital/year has a major impact on the outcomes? B) Question 9: Based on our calculations, would you definitely rule out one or more of the 3 hospitals if you had planned a TKA? C) Question 10: Currently, there is a minimum volume threshold for TKAs in Germany. This threshold means that a hospital has to do at least 50 TKAs/year to receive reimbursement for these surgeries. What would be your opinion if the minimum volume threshold were set to [number above the number from the 1st hospital] per year and [1st hospital] would no longer be allowed to perform this surgery? (*n* = 16). B = participant identification number; *n* = number of participants; TKA = total knee arthroplasty

Following the explanation, we asked the interviewees if the impact of the hospital TKA volumes on the outcomes was important to them. The differences in outcomes based on different hospital TKA volumes among the three hospitals were considered essential by eight interviewees; four were undecided, while a further four disagreed because they viewed the differences in outcomes as too small to be meaningful (Fig. 10 A). The majority of urban residents considered the differences in outcomes based on hospital volumes essential, while most of the rural residents were undecided or disagreed.

We subsequently asked if interviewees would reject one or more hospitals based on the results of our systematic review. These results contributed to hospital choice for six interviewees; five did not provide a clear statement for or against consideration of the results of the systematic review, while five disagreed because they viewed other factors such as the surgeon to be more important than the hospital TKA volume (Fig. 10 B). The majority of urban residents, but no rural resident, would choose a hospital based on the results of the systematic review. Most rural residents were either not sure or would not make their hospital choice based on the results of our systematic review.

Finally, we asked the interviewees about their opinion on raising the minimum volume threshold for TKA. Specifically, we asked them to consider the eventuality that due to raising the minimum volume threshold, the hospital nearest to their home was no longer allowed to perform TKA surgeries and, therefore, they would need to travel longer to receive TKA. Raising the minimum volume threshold for TKA was fully supported by six interviewees, partially supported by two, not supported by seven, while one was undecided (Fig. 10 C). The supporters favoured specialisation of hospitals regarding types of surgery. Others feared that surgeries would either be performed without medical necessity but only to increase the hospital TKA volume or that insufficient TKA volumes would cause hospitals to close, affecting access to healthcare, particularly in rural areas. The majority of urban residents, but only one rural resident, supported raising the minimum volume threshold for TKA. Most rural residents did not support raising the minimum volume threshold for TKA.

### Participant feedback

We presented a summary of our findings to one participant during the feedback session. The summary was discussed following the guide outlined in Table 3.

**Table 3:** Feedback session guide

Feedback topics	
**1)** Do you have comments on our interpretation of your answers?	
**2)** In your opinion, what are the consequences of these results?	
**3)** Since six other people in our study rule out one or more hospitals based on our scientific calculations, we are wondering:	
**a)** Where would you look for such quality data?	
**b)** What sources would you trust with such information?	
**c)** How should this information best be disseminated?	

Since the participant fully validated our interpretation of their own data, we did not seek feedback from others. According to the opinion of the participant, the most experienced surgeons and hospitals should perform TKAs. The participant suggested disseminating hospital quality information over the internet or through health magazines.

## Discussion

### Overall findings

Our study shows that potential patients believed that a hospital volume-outcome relationship exists for TKA. All participants considered that the quality of outcomes was more important than travel time to the hospital. However, when confronted with the results of a systematic review on this topic, only half of the participants stated that differences in outcomes related to the hospital TKA volume would have an important impact on their hospital choice for TKA surgery.

### Hospital volumes and the hospital volume-outcome relationship

Most participants in both phases of the current study (focus group and interviews) over- or underestimated hospital TKA volumes compared to the true values. The real median annual hospital TKA volumes in Germany (2009–2014) were 56 for the lowest, 195 for the middle, and 477 for the highest patient quintile [7], and the hospital with the highest TKA volume in 2018 performed 2,142 primary TKAs [39]. Our results demonstrate that hospital volumes were difficult to estimate. Despite these difficulties, our participants believed that a hospital volume-outcome relationship exists for TKA. Similarly, in other studies in the USA, participants believed that the number of times a surgeon performs rectal cancer surgery makes a difference to the outcomes [41], and candidates for hip or knee arthroplasty cared about hospital volume when deciding where to have surgery [26]. In Germany, patients ranked ‘volume of specified surgical procedures’ in 7th place out of 29 quality indicators [42], suggesting that hospital volume was perceived as important for outcome quality.

Nevertheless, our participants believed that other factors, including surgeon skills, surgeon volume and individual patient behaviour, could also influence the outcomes of TKA surgery. This approach seems logical considering that hospital volume by itself does not necessarily result in better health outcomes but rather is a proxy measure for various provider characteristics that could improve outcomes [9, 43], including infrastructure [9], compliance with evidence-based processes of care [9], and teamwork [44]. In addition, they mentioned that the number of surgeons per hospital was important when comparing hospital volumes.

### Hospital choice

#### Importance of hospital quality and travel time

Participants in both phases of the current study reported that the quality of the TKA was more important than travel time. This was especially true for those with rural residence, possibly, because this subgroup was used to longer travel times. Participants were prepared to travel a longer time within Germany or even within Europe to obtain the best possible outcomes. This approach is in line with a recent review of discrete choice experiments revealing a trend towards acceptance of greater travel times if the surgical risk in the local hospital increased [45]. In our study, some interviewees would base their hospital choice on the results of the systematic review on outcome quality rather than travel time to the hospital. However, several requisites for such a decision need to be met: information regarding hospital quality needs to exist, be available and be understandable to assist patients with decision-making [46, 47]. Patients must believe in the information and act on it [46].

#### Difficulties with hospital quality information

A Cochrane review found evidence that public dissemination of quality information may make little or no difference to the long-term healthcare utilisation by consumers [48]. Publicly available quality information played no role in the patients’ choice of a provider for hip and knee arthroplasty [27]. There are multiple reasons for these findings. First, in the case of Germany, hospitals have indeed been obliged to publish quality reports since 2005 [49], but awareness of such information is low among the general population [50]. Second, comparative health information is complex and can be misunderstood [51]. In our study, interviewees had questions regarding the personalised hospital risk sheets indicating that they required assistance with the comprehension of such data. While it was possible to address these questions in the context of the current study (during the interviews), patients typically have to understand the quality information without assistance. In sum, the usage of information regarding hospital quality is low among patients [27, 50, 52]. Possible reasons may be that patients trust their physicians to refer them to a hospital with expertise or expect the operating surgeons to tell them if the surgery was too complicated to be performed in a low-volume hospital [41] and therefore feel no need for more information [53].

#### Subjective factors for hospital choice

One precondition for choosing a hospital based on outcomes is that patients must act on hospital quality information as consumers [47]. This choice involves a rational decision to select the best-performing providers, which means that patients do not apply other criteria that might outweigh performance data [46]. However, patients do not necessarily make rational choices based on quality parameters alone [25, 54]. In line with previous research [25, 26, 55–57], participants in our study would consider a variety of subjective factors when deciding on a hospital for TKA surgery. Our results confirm the social influence [54] of trusted people such as relatives [26, 56], friends [25, 26, 55] and doctors [25, 26, 55, 56] on the hospital choice of patients. In line with previous research [23], reputation was very important to our participants for choosing a hospital. The results highlight the importance of social and communication skills of physicians [27, 42, 57]. As shown by others [26, 56], some of our participants would also search the internet.

In sum, hospital choice is influenced by many factors beyond quality alone, and patients have difficulties obtaining and correctly assessing hospital quality data. Therefore, it is unlikely that patients act as consumers and use quality information or that resulting competitive mechanisms lead to improved health outcomes [46], as suggested by the model of demand-driven healthcare derived from a neoliberal economic theory of market [19–21, 24]. However, our data show that potential patients were indeed interested in hospital quality information, similar to participants in other studies, who would consult such data if they were available [27, 58].

### Dissemination of hospital quality information

Based on our data, we focus on two methods for disseminating hospital quality information, while we acknowledge that other channels for sharing such information exist.

#### Referring physicians

Referring physicians, as trusted experts and possible multipliers, could increase both awareness and understanding of information on hospital quality and volumes if they discussed it with their patients. This suggestion is supported by the finding that, in contrast to low volume hospitals, patients with rectal cancer in high volume hospitals received the recommendation for such hospitals from their social networks, such as referring physicians or relatives with medical knowledge [41]. However, older studies show that not all physicians are aware of hospital quality reports (48% of surveyed German physicians in 2010 [59]) and that few use such reports for referral and counselling of patients (approximately 12–20%) [59–64]. It is possible that awareness and usage of hospital quality information has increased in recent years due to technological advancements in creating and disseminating information.

#### Internet

During the feedback session, our participant suggested that hospital quality information should be available on the internet from independent agencies (e.g., the federal statistics office) rather than on the websites of hospitals to assure its validity and objectivity. In Germany, such information is collated, for example, on the so-called “White List” [39] that is integrated into the new health information portal published by the Federal Ministry of Health [65]. Future studies need to evaluate whether these sources affect the awareness and usage of healthcare quality information by physicians [63] and patients in Germany.

In general, the public (worldwide) has the right to know how well their healthcare system works, irrespective of whether they use the information. Reported outcomes have to be relevant to end users: studies have shown that functional outcomes are more important to patients than mortality [54], emphasising the structural collection and publication of patient-relevant outcome measurements. At the same time, public reporting can also induce dysfunctional responses such as cream skimming, teaching to test and manipulating data-coding practices [66].

### Minimum volume thresholds

Minimum volume thresholds aim to guarantee a minimum level of quality but may increase travel time for some patients [15]. Therefore, empirical data need to prove that higher hospital volumes are associated with better outcomes. Half of the interviewees supported raising the minimum volume threshold for TKA despite theoretically increased travel times to receive TKA surgery. On the one hand, this approach is logical if participants believe that a hospital volume-outcome relationship exists and consider outcome quality to be important [45]. On the other hand, it is surprising since policy-makers consider local healthcare to be very important [67], and German citizens have protested against planned closure of hospitals in the past [68, 69]. Closure of hospitals as a consequence of the minimum volume threshold could limit the availability of healthcare services, especially in rural areas, and thus was also feared by some of our participants. In particular, participants with rural residence did not support raising the minimum volume threshold for TKA. There are two explanations for this finding: on the one hand, the patients with rural residence may be more affected by longer travel times if TKA surgery was centralised in metropolitan areas. On the other hand, most participants with rural residence did not consider the impact of the hospital TKA volume on the outcomes to be important and would not base their hospital choice on the results of our systematic review.

However, research has shown that 99% of patients affected by the current minimum volume threshold of 50 TKAs per year in Germany could reach a hospital performing this number of TKAs within 34 min [70], and this travel time was acceptable for all our participants. Some of our participants believed that a higher minimum volume threshold could increase the number of TKAs performed without medical necessity. This finding is alarming because it demonstrates that participants do not fully trust in being treated for medical reasons and worry that economic reasons play a major role in a healthcare system that provides economic incentives to increase procedure volumes. In contrast, some experts argue that large increases in minimum volume thresholds would reduce the risk of surgeries being performed without medical necessity: if the threshold were very high and thus unreachable for low volume hospitals, there would be no incentive for a few additional procedures [71]. However, if the threshold remained low (50 TKAs), this threshold may be reached easily with a few additional TKAs, meaning that the risk for TKAs without medical necessity increases [71]. Our participants also worried about anonymity in larger hospitals with multiple physician teams and preferred the concept of a surgeon individually assigned from admission to follow-up treatment. Indeed, others [72] showed that hospital size had a negative impact on patient satisfaction. Assuming that higher TKA volumes will usually be reached in larger and more specialised hospitals, measures to increase patient satisfaction (in addition to hospital quality performance) have to be implemented.

### Strengths and limitations

To our knowledge, this is the first study that used personalisation of the results of a systematic review for the evaluation of patient preferences. We used personalised hospital risk sheets to discuss hospital choice with potential patients. We believe our approach could be applied to future systematic reviews if topic and context allow it, for instance, in healthcare planning. Other authors conclude that patient and public involvement may have a positive effect on implementation, although effectiveness of public involvement is unclear [73].

Our study has several limitations. First, our sample was relatively small in view of the complexity of the topic, and we did not reach data saturation, meaning that new topics were mentioned in the last interviews. The majority of participants were highly educated and resided in a densely populated federal state of Germany (North Rhine-Westphalia) with a large choice of local hospitals, although we differentiated between urban and rural residents. While others used a similar sample size when investigating patient preferences for hospitals [27], more participants with heterogeneous characteristics should be involved in future research on this topic. Furthermore, hospital choice and opinion on minimum volume thresholds should also be assessed for people residing in less densely populated areas with lower access to local hospitals.

Second, the sample was selected from the general population reporting knee problems, but our participants were not (yet) facing a real hospital choice for TKA, meaning that their theoretical choices may not align with their actual behaviour in practice [25]. In fact, studies showed that only a proportion of patients actively decided themselves on a provider [25] (63% of hospitalised patients in Germany in one study), and, for example, doctors or relatives made the decision [56]. Therefore, we do not know if the participants who stated that they would base their hospital choice on the results of our systematic review would actually do so when faced with real hospital choice for TKA in the future.

Third, due to contact restrictions during the SARS-CoV-2 pandemic in 2020, we performed individual interviews instead of focus groups to discuss the results of our systematic review with the participants. Others [74] have reported that while more concepts were identified during focus groups compared to individual interviews, the type of information obtained with the two methods was similar. It would also be interesting to discuss the influence of the results of a systematic review on hospital choice in group contexts. Group discussions may be more suitable to explore rural-urban differences in hospital choice for TKA.

Fourth, the outcomes from our systematic review presented to the participants were limited to harms outcomes that were reported in the literature, although others have suggested preferentially reporting functional or patient-reported outcomes, which are more important to patients than mortality [54]. Our personalised hospital risk sheets did not include complex statistical information, such as confidence intervals or risk of bias assessments. Instead we used simple pictograms to incorporate the outcomes of our dose-response meta-analysis in the risk sheets. This approach was effective at generating a lively discussion with potential patients during the individual interviews, indicating that the information was comprehensible when the interviewer was able to answer the questions interviewees had.

One strength of our study is that we validated our findings using an additional feedback session with one participant as recommended by the COREQ guidelines [30]. We did not return the full interview transcript to the participants because changes in transcripts made by the responders did not affect the findings in previous studies [75]. Instead, selective member checks provided opportunities for comments and feedback [75] and allowed us to discuss access to information on hospital quality with our participant.

## Conclusions

Potential TKA patients believe that a hospital volume-outcome relationship exists for TKA. While hospital preference is based mainly on subjective factors, some potential patients would consider scientific evidence when making their choice. However, patients find hospital quality data difficult to obtain and assess. Relevant information sources should be simplified to increase comprehensibility and disseminated more widely. Policy makers and physicians should consider the patient perspectives when deciding on the minimum volume threshold or recommending hospitals for TKA, respectively.

## Supplementary Information

**Additional file 1.** COREQ checklist.

**Additional file 2.** Research team and reflexivity.

**Additional file 3.** Coding tree focus group.

**Additional file 4.** Coding tree interviews.

**Additional file 5.** Selected quotes translation.

## Data Availability

The datasets used and/or analysed during the current study are available from the corresponding author on reasonable request.

## Abbreviations

B: participant identification number
TKA: Total knee arthroplasty
COREQ: Consolidated Criteria for Reporting Qualitative Research recommendations
