# Supplementary material for: Perspective of potential patients on the hospital volume-outcome relationship and the minimum volume threshold for total knee arthroplasty: a qualitative focus group and interview study
Source: BMC Health Serv Res. 2021 Jul 2;21:633. doi: 10.1186/s12913-021-06641-8 (PMC8249216; doi:10.1186/s12913-021-06641-8)
Supplement: Supplementary file 1 — Additional file 1. COREQ checklist. [file 12913_2021_6641_MOESM1_ESM.pdf]

Additional file 1: COREQ checklist

Perspective of potential patients on the hospital volume-outcome relationship and the minimum volume threshold for total knee arthroplasty: A qualitative focus group and interview study

Authors:

M.Sc. Charlotte M. Kugler charlotte.kugler@uni-wh.de (1), PhD Karina K. De Santis desantis@leibniz-bips.de (1), MPH Tanja Rombey tanja.rombey@uni-wh.de (1), PhD Kaethe Goossen kaethe.goossen@uni-wh.de (1), M.Sc. Jessica Breuing jessica.breuing@uni-wh.de (1), M.Sc. Nadja Koensgen nadja.koensgen@uni-wh.de (1), Dr. Tim Mathes tim.mathes@uni-wh.de (1), Simone Hess simone.hess@uni-wh.de (1), Dr. René Burchard rene.burchard@uni-wh.de (2, 3, 4), Dr. Dawid Pieper dawid.pieper@uni-wh.de (1)

(1) Institute for Research in Operative Medicine, Witten/Herdecke University, Ostmerheimer Str. 200, 51109 Cologne, Germany

(2) Department of Trauma Surgery and Orthopaedics, Lahn-Dill-Kliniken, Rotebergstr. 2, 35683 Dillenburg, Germany

(3) Department of Health, Witten/Herdecke University, Alfred-Herrhausen-Straße 50, 58448 Witten, Germany

(4) School of Medicine, Univerity of Marburg, Baldingerstraße, 35032 Marburg, Germany

Corresponding author: Charlotte M Kugler, charlotte.kugler@uni-wh.de, Tel: +49 221 9895742

Consolidated criteria for reporting qualitative studies (COREQ): 32-item checklist [1]

No. Item	Reported in detail:

**Domain 1: Research team and reflexivity**	
Personal Characteristics	
1. Interviewer/ facilitator	2.2 / Figure 1
2. Credentials	Authors
3. Occupation	Additional file 2
4. Gender	Additional file 2
5. Experience and training	Additional file 2
Relationship with participants	
6. Relationship established	2.1; Additional file 2
7. Participant knowledge of the interviewer	2.1, Figure 1, Additional file 2
8. Interviewer characteristics	Additional file 2
**Domain 2: study design**	
Theoretical framework	
9. Methodological orientation and Theory	2.3, Figure 2
Participant slection	
10. Sampling	2.1
11. Method of approach	2.1
12. Sample size	3.1, Table 2
13. Non-participation	3.1, Table 2
Setting	
14. Setting of data collection	2.3, Figure 1
15. Presence of non-participants	2.3, Figure 1
16. Description of sample	3.1, Table 2
Data collection	
17. Interview guide	2.2, Table 1
18. Repeat interviews	2.1
19. Audio/visual recording	2.1, Figure 1
20. Field notes	2.1, Figure 1
21. Duration	2.1, Figure 1
22. Data saturation	4.6
23. Transcripts returned	2.4 Figure 2
**Domain 3: analysis and findings**	
Data analysis	
24. Number of data coders	2.4 Figure 2
25. Description of the coding tree	Additional files 3 & 4
26. Derivation of themes	2.4 Figure 2
27. Software	2.4 Figure 2
28. Participant checking	2.5, 3.4
Reporting	
29. Quotations presented	3.2, 3.3, Figure 3-7, Figure 10
30. Data and findings consistent	Results
31. Clarity of major themes	Figure 3-7, 10 (Results)
32. Clarity of minor themes	Figure 3-7, 10 (Results)

1. Tong A, Sainsbury P, Craig J. Consolidated criteria for reporting qualitative research (COREQ): a 32-item checklist for interviews and focus groups. Int J Qual Health Care. 2007;19(6):349-57.doi:10.1093/intqhc/mzm042
