# Supplementary material for: Perspective of potential patients on the hospital volume-outcome relationship and the minimum volume threshold for total knee arthroplasty: a qualitative focus group and interview study
Source: BMC Health Serv Res. 2021 Jul 2;21:633. doi: 10.1186/s12913-021-06641-8 (PMC8249216; doi:10.1186/s12913-021-06641-8)
Supplement: Supplementary file 2 — Additional file 2. Research team and reflexivity. [file 12913_2021_6641_MOESM2_ESM.pdf]

Additional file 2: Research team and reflexivity

Perspective of potential patients on the hospital volume-outcome relationship and the minimum volume threshold for total knee arthroplasty: A qualitative focus group and interview study

Authors:

M.Sc. Charlotte M. Kugler charlotte.kugler@uni-wh.de (1), PhD Karina K. De Santis desantis@leibniz-bips.de (1), MPH Tanja Rombey tanja.rombey@uni-wh.de (1), PhD Kaethe Goossen kaethe.goossen@uni-wh.de (1), M.Sc. Jessica Breuing jessica.breuing@uni-wh.de (1), M.Sc. Nadja Koensgen nadja.koensgen@uni-wh.de (1), Dr. Tim Mathes tim.mathes@uni-wh.de (1), Simone Hess simone.hess@uni-wh.de (1), Dr. René Burchard rene.burchard@uni-wh.de (2, 3, 4), Dr. Dawid Pieper dawid.pieper@uni-wh.de (1)

(1) Institute for Research in Operative Medicine, Witten/Herdecke University, Ostmerheimer Str. 200, 51109 Cologne, Germany

(2) Department of Trauma Surgery and Orthopaedics, Lahn-Dill-Kliniken, Rotebergstr. 2, 35683 Dillenburg, Germany

(3) Department of Health, Witten/Herdecke University, Alfred-Herrhausen-Straße 50, 58448 Witten, Germany

(4) School of Medicine, Univerity of Marburg, Baldingerstraße, 35032 Marburg, Germany

Corresponding author: Charlotte M Kugler, charlotte.kugler@uni-wh.de, Tel: +49 221 9895742

Characteristic	Description
Credentials	All senior authors (KG, KDS, TM, RB and DP) hold doctoral degrees. CK, JB, TR, and NK hold master degrees and TR, JB and NK are currently doctoral candidates. SH is trained as a medical-laboratory assistant.

Occupation	CK, KDS, KG, JB, NK, TM, SH and DP work as researchers at the Witten/Herdecke University. RB works as leading physician in an orthopaedic clinic.
Gender	CK, KDS, TR, KB, JB, NK, SH are female, TM, RB, DP are male.
Experience and training	CK, KDS, KG, JB, NK, TM, DP are health or life scientists, TM is also statistician, RB is an orthopaedist. SH is an information specialist. KG, KDS, TM, RB and DP have co-authored multiple publications focusing on quantitative or qualitative research methods in health sciences. JB has previous experience in qualitative studies and conducting focus groups as well as interviews. CK has previous experience in conducting and analysing interviews.
Relationship between participants and researchers	There was no relationship between the moderator and the participants of the focus group (JB) (Phase 1) nor between the interviewer and the interviewees. There was a relationship between one participant of the focus group and the co-moderator (TR) and between interviewees and other researchers (KG, NK, DP) since they were recruited by word of mouth.
Participant knowledge of the interviewer	Participants did not know the moderator / interviewer. They knew they were researchers at Witten/Herdecke University. The participants were given the information that the project was about the hospital TKA volume-outcome relationship and minimum volume thresholds and that the aim of the study was to gain insight into personal need and preferences of potential patients on this topic.
Interviewer characteristics	The main interest of JB and TR in the topic was based on previous research on the volume-outcome relationship.
