# Supplementary material for: Perspective of potential patients on the hospital volume-outcome relationship and the minimum volume threshold for total knee arthroplasty: a qualitative focus group and interview study
Source: BMC Health Serv Res. 2021 Jul 2;21:633. doi: 10.1186/s12913-021-06641-8 (PMC8249216; doi:10.1186/s12913-021-06641-8)
Supplement: Supplementary file 3 — Additional file 3. Coding tree focus group. [file 12913_2021_6641_MOESM3_ESM.pdf]

Additional file 3: Coding tree focus group

Perspective of potential patients on the hospital volume-outcome relationship and the minimum volume threshold for total knee arthroplasty: A qualitative focus group and interview study

Authors:

M.Sc. Charlotte M. Kugler charlotte.kugler@uni-wh.de (1), PhD Karina K. De Santis desantis@leibniz-bips.de (1), MPH Tanja Rombey tanja.rombey@uni-wh.de (1), PhD Kaethe Goossen kaethe.goossen@uni-wh.de (1), M.Sc. Jessica Breuing jessica.breuing@uni-wh.de (1), M.Sc. Nadja Koensgen nadja.koensgen@uni-wh.de (1), Dr. Tim Mathes tim.mathes@uni-wh.de (1), Simone Hess simone.hess@uni-wh.de (1), Dr. René Burchard rene.burchard@uni-wh.de (2, 3, 4), Dr. Dawid Pieper dawid.pieper@uni-wh.de (1)

(1) Institute for Research in Operative Medicine, Witten/Herdecke University, Ostmerheimer Str. 200, 51109 Cologne, Germany

(2) Department of Trauma Surgery and Orthopaedics, Lahn-Dill-Kliniken, Rotebergstr. 2, 35683 Dillenburg, Germany

(3) Department of Health, Witten/Herdecke University, Alfred-Herrhausen-Straße 50, 58448 Witten, Germany

(4) School of Medicine, Univerity of Marburg, Baldingerstraße, 35032 Marburg, Germany

Corresponding author: Charlotte M Kugler, charlotte.kugler@uni-wh.de, Tel: +49 221 9895742

Dimension	Subkategorie	Sub-Subkategorie	Definition	Beispiel	Kodier Regel
1. Gründe für Knie-TEP	1.1 Sportunfälle		Teilnehmer*innen halten Sportunfälle als eine mögliche Ursache für die Notwendigkeit einer Knie-TEP.	„Skifahrer, Motorradfahrer"	Wörter/ Sätze kodieren, die als Grund für eine Knie-TEP gesehen werden.
	1.2 Krebsfälle		Teilnehmer*innen halten Krebsdiagnosen als eine mögliche Ursache für die Notwendigkeit einer Knie-TEP.	„Es können aber auch Krebsfälle sein."	Wörter/ Sätze kodieren, die als Grund für eine Knie-TEP gesehen werden.
	1.3 Schmerzen		Teilnehmer*innen halten Schmerzen als eine mögliche Ursache für die Notwendigkeit einer Knie-TEP.	„Ich glaube, ein großer Faktor ist der Ruheschmerz."	Wörter/ Sätze kodieren, die als Grund für eine Knie-TEP gesehen werden.
	1.4 Verschleiß		Teilnehmer*innen halten Verschleiß als eine mögliche Ursache für die Notwendigkeit einer Knie-TEP.	„Also, ich nehme an, die meisten OPs ist das der Verschleiß."	Wörter/ Sätze kodieren, die als Grund für eine Knie-TEP gesehen werden.
2. Durchschnittliche Klinik	2.1 Abhängigkeitsfaktoren		Teilnehmer*innen denken, dass die durchschnittliche Fallzahl eines Krankenhauses von Faktoren, wie z. B. der Größe des Krankenhauses abhängig ist.	„Also, es kommt wahrscheinlich auch auf die Größe des Krankenhauses an."	Satz / Absatz kodieren, falls vorhanden
	2.2 Berechnung		Teilnehmer*innen erklären, wie er/sie die Fallzahl in einer durchschnittlichen Klinik geschätzt hat: anhand der Mindestmenge, anhand 200.000 Operationen im Jahr oder anhand einer Schätzung pro Woche oder pro Tag.	„Ich denke, täglich zwei drei. Und das dann malnehmen."	Äußerung (Satz/Absatz) kodieren falls vorhanden.
	2.3 Zahl Durchschnitt		geschätzte Fallzahl durch Teilnehmer*innen in einer durchschnittlichen Klinik	„100"	nur die Zahl kodieren
3. Klinik geringe Fallzahl	3.1 Zahl gering		geschätzte Fallzahl durch Teilnehmer*innen in einem Krankenhaus mit einer geringen Anzahl an TEPs	„50"	nur die Zahl kodieren
4. Klinik hohe Fallzahl	4.1 Zahl hoch		geschätzte Fallzahl durch Teilnehmer*innen in einem Krankenhaus mit einer hohe Anzahl an TEPs	„1000" / „500-1000"	nur die Zahl kodieren
	4.2 Abhängigkeitsfaktor		Teilnehmer*in denkt, dass eine hohe Fallzahl je nach Art der Klinik unterschiedlich ist.	„Es kommt wahrscheinlich auch auf die Größe des Krankenhauses an."	Satz / Absatz kodieren
	5.1 klare Zustimmung		Teilnehmer*innen äußern klare Zustimmung ohne Einschränkung.	„Ja. Würde ich voll zustimmen."<br>„Ich glaube, dass das stimmt."	Äußerung kodieren (Satz / Absatz)<br>Begründung als Subkategorie kodieren
		5.1.1 Routine/Erfahrung	Routine (positiv & negativ), Erfahrung, Erfahrungswerten wird als Einflussfaktor für das Ergebnis einer Operation genannt.	„Würde man erwarten, weil ja die Routine da sicher auch eine große Rolle spielt dann. Wenn also Ärzte das häufiger machen, dann kennen sie sich vermutlich besser aus als jemand, der das nur ab und zu macht."<br>„Also je mehr Erfahrungen ein Krankenhaus damit macht desto besser werden auch die Ergebnisse."<br>„(...) weil man eben im Geschäft ist."	Wörter oder Sätze kodieren, die Routine als Erklärungsfaktor belegen. Wenn Routine auch negativ gesehen wird, zusätzlich "Erfahrung/Routine negativ" kodieren. Mehrere Faktoren können kodiert werden.
		5.1.2 spezifische Fortbildungen	Teilnehmer*in vermutet, dass spezifischere Fortbildungen belegt werden, wenn die Fallzahl steigt.	„Ich hoffe auch, dass diejenigen, die das oft machen, auch interessiert sind an Neuerungen, an Fortbildungen. Was gibt es neu, was kann man noch verbessern. Und wenn das sehr spezialisiert ist, dann kann man das ja auch speziell weitergeben."	Wörter oder Sätze kodieren, die spezifische Fortbildungen als Erklärungsfaktor belegen. Mehrere Faktoren können kodiert werden.
		5.1.3 Ausstattung des Krankenhauses	Als Einflussfaktor des Ergebnisses einer Operation wird die Ausstattung des Krankenhauses genannt: Mit mehr Fällen ist auch die Ausstattung des Krankenhauses besser.	„Wenn natürlich nur Umsatz ist, denke ich einmal auch, dass auch die nötigen Instrumente da sind, die eventuell, wenn es nur geringfügig gemacht wird in einem Krankenhaus, einfach nicht da sind. (...) Also, da sind sie einfach besser ausgestattet."	Wörter oder Sätze kodieren, die die Ausstattung des Krankenhauses als Erklärungsfaktor belegen. Mehrere Faktoren können kodiert werden.
		5.1.4 Spezialisierung	Teilnehmer*in vermutet, dass sich das Ergebnis durch eine steigende Spezialisierung verbessert.	„Also ich denke schon, dass wenn es viele Knie-OPs sind und es ist sagen wir mal die Leute sich darauf spezialisiert haben, denke ich schon, dass die Ergebnisse vermutlich im Schnitt besser sind als im anderen Krankenhaus, wo man von Hüfte über Kopf über Zehen alles machen muss, weil es einfach anfällt, ja."	Wörter oder Sätze kodieren, die die Spezialisierung als Erklärungsfaktor belegen. Mehrere Faktoren können kodiert werden.

Dimension	Subkategorie	Sub-Subkategorie	Definition	Beispiel	Kodier Regel
		5.1.5 Umgang mit Komplikationen	Teilnehmer*in nimmt an, dass der routinierte Umgang mit Komplikationen das Ergebnis der Operation beeinflusst.	„Und wenn mehr durchgeführt werden, gehe ich einmal davon aus, dass die Klinik auch entsprechend größer ist, wenn es jetzt, sagen wir mal, eine Durchschnittsklinik ist. Also, auf das Knie spezialisiert ist. Und von daher gibt es dann auch noch eine bessere Versorgung, falls dann irgendetwas auftreten sollte."	Wörter oder Sätze kodieren, die den Umgang mit Komplikationen als Erklärungsfaktor belegen. Mehrere Faktoren können kodiert werden.
5. Reaktion: Einfluss der Fallzahl auf das Ergebnis / Erklärung des Zusammenhangs bzw. Nennung von anderen Einflussfaktoresn	5.2 Zustimmung mit Einschränkung		Teilnehmer*innen äußern Zustimmung mit Einschränkung.	„Kommt darauf an. Wenn es eine hohe Zahl ist, also, wenn wirklich die Routine da ist, dann ist es sicherlich so, dass die Erfahrung letztlich zur Qualität beiträgt, zur höheren. Wenn es allerdings ohnehin, sagen wir einmal, eine spezialisierte Klinik ist, muss das nicht unbedingt sein. Also, ich denke einmal, ganz hoch muss es nicht sein, aber es sollt eine gewisse Routine da sein."	Äußerung + Begründung kodieren (Satz / Absatz). Einschränkung bzw. Faktoren, die das Ergebnis einer Operation beeinflussen, einzeln als Subsubkategorie kodieren.
		5.2.1 Routine / Erfahrung negativ	Routine wird dabei (auch) negativ bewertet.	„Von daher würde man sagen, dass man, wenn man mehr Übung hat, besser wird. Aber es kann natürlich auch genauso gut dazu führen, dass, wenn es zu viele sind, kommt da die Routine zu stark heraus. Man wird zu routiniert und achtet nicht mehr auf jedes Detail. "	Wörter oder Sätze kodieren, die Routine als **negativen Einfluss** belegen. Mehrere Faktoren können kodiert werden.
		5.2.2 Eignung Ärzt*in	Teilnehmer*in äußert, dass die Eignung der Ärzt*innen ebenfalls einen Einfluss hat.	„Es kann auch jemand sein, der noch nicht so lange Erfahrung hat, dass der einfach ein Händchen hat."	Einschränkung kodieren (Satz / Absatz), mehrere Einschränkungen möglich.
		5.2.3 Narkose	Teilnehmer*in meint, dass die Narkose einen Einfluss auf den Gesundheitszustand hat und dadurch auch das Ergebnis der Knie-TEP beeinfluss wird.	„Was für einen alten Menschen auch Probleme macht, ist die Narkose. Mein Vater musste ja dann in sieben Monaten viermal operiert werden. (...) Wenn sie ängstlich sind, bewegen sie sich nicht, auch, wenn es nicht wehtut."	Wörter oder Sätze, die diesen Einflussfaktor belegen, kodieren.
		5.2.4 Reha/Nachsorge	Teilnehmer*in denkt, dass die Reha / Nachsorge nach der OP einen Einfluss auf das Ergebnis der Operation hat.	„Man könnte auch zum Beispiel an die Nachsorge im engeren Sinne denken. Zum Beispiel das Pflegepersonal, die Physiotherapeuten, die sie unmittelbar danach noch bekommen."	Wörter oder Sätze, die diesen Einflussfaktor belegen, kodieren.
		5.2.5 Qualität der Prothese	Teilnehmer*in denkt, dass die Qualität der Prothese einen Einfluss auf das Ergebnis der Operation hat.	„Die Qualität der Ersatzteile. Ich weiß nicht, ob es da verschiedene Qualitätsmerkmale gibt."	Wörter oder Sätze, die diesen Einflussfaktor belegen, kodieren.
		5.2.6 Verhalten der Patient*innen	Teilnehmer*in denkt, dass auch das Verhalten der Patient*innen (nach der OP) einen Einfluss auf das Ergebnis hat.	„(...) ‚Bitte rauchen Sie nicht nach der OP.' und das erste, was die Leute machen, gehen raus vor das Krankenhaus und rauchen. Das beeinflusst natürlich auch insgesamt den Genesungsprozess und viele andere Sachen. Also das Verhalten einfach des Patienten danach. Und da kann ja der Operateur noch so gut sein."	Wörter oder Sätze, die diesen Einflussfaktor belegen, kodieren.
		5.2.7 Team	Teilnehmer*in meint, dass das Team Einfluss auf das Ergebnis einer Operation hat.	„Und der macht das ja auch nicht allein. Also es ist ja nicht so, dass der da allein steht. Sondern, da haben Sie ja Leute drumherum, die aufpassen."	Wörter oder Sätze, die diesen Einflussfaktor belegen, kodieren.
	5.3 klare Ablehnung		Teilnehmer*in äußert klare Ablehnung.	„Nein, ich denke nicht, dass das stimmt."	Äußerung + ggf. Begründungkodieren (Satz / Absatz).
	6.1 "Gute" Zahl		Fallzahl, die als überzeugend angegeben wird, um sich in einem Krankenhaus mit dieser Fallzahl operieren zu lassen	„1000"	Zahl + Einheit kodieren.
6. Überzeugende Fallzahl	6.2 Einschränkung: Empfehlung wichtiger als Fallzahl		Teilnehmer*in gibt an, dass statt der Fallzahl die Empfehlung durch Bekannte wichtiger ist, um sich für oder gegen ein Krankenhaus zu entscheiden.	„Ja, würde mich auch beeinflussen lassen von Freunden, die da schon waren, wo es gut gelaufen ist. Von Freunden, Verwandten, oder -"	Einschränkung kodieren.
	6.3 Einschränkung: abhängig von Fallzahl in Umgebung		Teilnehmer*in gibt an, dass die Fallzahl, die als überzeugend wahrgenommen wird von den Fallzahlen der Krankenhäuser in der Umgebung abhängt.	„Ich würde das gar nicht so an einer Zahl festmachen. Ich würde mir Vergleichswerte in der Umgebung suchen, in dem [Name einer Stadt] Raum, und da schauen, was es da so gibt und wahrscheinlich tendenziell eher zu der gehen die am meisten hat."	Einschränkung kodieren.

Dimension	Subkategorie	Sub-Subkategorie	Definition	Beispiel	Kodier Regel
7. Distanz zur nächsten Klinik			Zahl in min / km, die als Distanz zur nächstgelegenen Klinik der Teilnehmer*innen angegeben wird.	„10 min zu Fuß"	Zahl + Einheit kodieren.
8. Anzahl Kliniken in Umgebung (Stadt)			Die Anzahl der Kliniken in der näheren Umgebung (innerhalb der Großstadt, in der die Fokusgruppe stattgefunden hat, was einem Umkreis von ca. 20 km entspricht) der Teilnehmer*innen	„10 Minimum"<br>„Ich glaube, dass sind auch noch zwei-drei Krankenhäuser"	Satz bzw. Zahl + Einheit kodieren.
9. Distanz Qualität	9.1 klare Zustimmung		Teilnehmer*innen äußern, dass sie (ohne Einschränkung) eine weitere Anfahrt für ein besseres Ergebnis einer Operation auf sich nehmen würden.	„Ja, da würde ich zustimmen."	Äußerung + Begründungkodieren (Satz / Absatz). Genannte Begründung als Subkategorie einfügen und ebenfalls kodieren.
	9.2 Zustimmung mit Einschränkung		Teilnehmer*innen äußern, dass sie für ein besseres Ergebnis eine weitere Fahrt auf sich nehmen würden, allerdings mit Einschränkung(en).	„Ja, also ich denke, das ist auch noch von anderen Kriterien abhängig."<br>„Also wenn ich sage mal nicht eine Routinesache ist, würde ich definitiv immer nach einer Spezialklinik oder Spezialkrankenhaus gucken."	Äußerung + Begründung kodieren (Satz / Absatz). Einschränkung bzw. Faktoren, die das Ergebnis einer Operation beeinflussen, einzeln als Subsubkategorie kodieren.
		9.2.1 Nachuntersuchungen im Krankenhaus	Teilnehmer*innen äußern Zustimmung für eine weitere Anfahrt, falls keine Nachuntersuchungen in dem weiter entfernten Krankenhaus, in dem die OP stattgefunden hat, anstehen.	„Es sei denn, es ist wirklich mit Nachuntersuchungen im Operationsort verbunden, aber wenn das jetzt ein einmaliger Eingriff ist."	Einschränkung kodieren (Satz / Absatz)
	9.3 klare Ablehnung		Teilnehmer*innen würden für ein bessere Ergebnis definitiv nicht weiter fahren.	„Nein, ich möchte in der Nähe behandelt werden."	Äußerung + ggf. Begründung kodieren (Satz / Absatz).
10. max Distanz in km / min	10.1 Zahl		geschätzte Entfernung + Einheit, die die Teilnehmer*innen bereit sind zu fahren	„100 km" / „7 Stunden"	Zahl + Einheit kodieren. Falls Begründung / Einschränkung genannt wird, diese als Subkategorie mitkodieren.
	10.2 Einschränkung der Distanz		Teilnehmer*innen schränken die von anderen Teilnehmer*innen genannte Distanz wieder ein.	„Also [Name einer Stadt, ca. 600 km Entfernung] ist ja jetzt schon weit würde ich sagen, da gibt es bestimmt noch was dazwischen, würde ich sagen."	Satz / Absatz mit Einschränkung der Distanz kodieren.
		10.2.1 Besuchsmöglichkeit bei älteren Menschen	Teilnehmer*innen geben zu Bedenken, dass ältere Menschen Besuch brauchen (für Versorgung mit Wäsche, Gespräch mit Ärzt*innen) und deshalb die Distanz ggf. eingeschränkt wird.	„Also wenn ich jetzt an meine Mutter denke, da wäre es sicherlich schon ein Thema, weil [sie] das nicht alleine machen könnte. Und da muss [man] natürlich auf der anderen Seite überlegen, wie die Angehörigen auch integrieren [werden] könne[n]. Wäsche vorbeibringen können und sowas."	Satz / Absatz für den Einschränkungsfaktor Besuch kodieren.
	10.3 Qualität wichtigster Faktor		Teilnehmer*innen erwiedern auf die Einschränkungen, dass die Qualität der wichtigste Faktor für die Wahl eines Krankenhauses ist.	„Qualität ist das Wichtigste."	Satz/Absatz kodieren, der diese Aussage belegt.

Dimension	Subkategorie	Sub-Subkategorie	Definition	Beispiel	Kodier Regel
Regeln:	Bei Subsubkategorien auch Subkategorie mitkodieren.<br>Fragliche Fälle mit Memo versehen, um zu besprechen. Können schon mit der Kategorie kodiert werden, wohin die Tendenz der Coderin geht.<br>Generell eher ganze Sätze als einzelne Wörter kodieren, wenn der Zusammenhang für die Nutzung als Zitat relevant ist.<br>Wenn die Fokusgruppenleiterin eine Zusammenfassung über eine Kategorie äußert und die Teilnehmer*innen zustimmen als Consens-Unterkategorie kodieren.				

19
