# Supplementary material for: Perspective of potential patients on the hospital volume-outcome relationship and the minimum volume threshold for total knee arthroplasty: a qualitative focus group and interview study
Source: BMC Health Serv Res. 2021 Jul 2;21:633. doi: 10.1186/s12913-021-06641-8 (PMC8249216; doi:10.1186/s12913-021-06641-8)
Supplement: Supplementary file 4 — Additional file 4. Coding tree interviews. [file 12913_2021_6641_MOESM4_ESM.pdf]

Additional file 4: Coding tree interviews

Perspective of potential patients on the hospital volume-outcome relationship and the minimum volume threshold for total knee arthroplasty: A qualitative focus group and interview study

Authors:

M.Sc. Charlotte M. Kugler charlotte.kugler@uni-wh.de (1), PhD Karina K. De Santis desantis@leibniz-bips.de (1), MPH Tanja Rombey tanja.rombey@uni-wh.de (1), PhD Kaethe Goossen kaethe.goossen@uni-wh.de (1), M.Sc. Jessica Breuing jessica.breuing@uni-wh.de (1), M.Sc. Nadja Koensgen nadja.koensgen@uni-wh.de (1), Dr. Tim Mathes tim.mathes@uni-wh.de (1), Simone Hess simone.hess@uni-wh.de (1), Dr. René Burchard rene.burchard@uni-wh.de (2, 3, 4), Dr. Dawid Pieper dawid.pieper@uni-wh.de (1)

(1) Institute for Research in Operative Medicine, Witten/Herdecke University, Ostmerheimer Str. 200, 51109 Cologne, Germany

(2) Department of Trauma Surgery and Orthopaedics, Lahn-Dill-Kliniken, Rotebergstr. 2, 35683 Dillenburg, Germany

(3) Department of Health, Witten/Herdecke University, Alfred-Herrhausen-Straße 50, 58448 Witten, Germany

(4) School of Medicine, Univerity of Marburg, Baldingerstraße, 35032 Marburg, Germany

Corresponding author: Charlotte M Kugler, charlotte.kugler@uni-wh.de, Tel: +49 221 9895742

Dimension	Subkategorie	Sub-Subkategorie	Definition	Beispiel	Kodier Regel
	1.1 Verständnisfragen / Abhängigkeitsfaktoren		Interviewte*r hat Fragen, was eine durchschnittliche Klinik ist bzw. denkt, dass die Fallzahl von anderen Faktoren, wie der Größe des Krankenhauses abhängig ist.	„Eine ganz normale Klinik?"<br>„Ja, was meinen Sie jetzt mit Krankenhaus? Es gibt große, es gibt kleine. Relativ gesehen, jetzt bezogen auf die Betten?"	Frage(n) /Anmerkungen kodieren
	1.2 Schwierigkeit		Interviewte*r äußert Schwierigkeiten die Zahlen einzuschätzen oder braucht lange für die Einschätzung.	„Oh je, das ist natürlich eine schwierige Frage (...) Weiß ich nicht, kann ich nicht sagen." / "Puh" / Lange Anzahl an Sek Pause	Äußerung (Satz oder Sätze) / Sekundenanzahl kodieren
	1.3 Berechnung		Interviewte*r erklärt, wie er/sie die Fallzahl in einer durchschnittlichen Klinik geschätzt hat: anhand der Mindestmenge, anhand 200.000 Operationen im Jahr oder anhand einer Schätzung pro Woche oder pro Tag.	siehe Subkategorien	Äußerung (Satz/Absatz) kodieren falls vorhanden. Die spezifische Berechnung als Subsubkategorie ebenfalls kodieren.
1. Durchschnittliche Klinik		1.3.1 Mindestmenge	Interviewte*r schätzt die Anzahl der OPs pro Krankenhaus aufgrund des Wissens über die Mindestmenge von 50 Knie-TEPs / Jahr.	„Ja, da war doch in diesem einen Infoschreiben, da stand doch auch so eine Zahl. Dass da mindestens fünfzig durchgeführt werden müssen. Also wenn es mindestens sind, dann müssen es schon einmal mehr als fünfzig sein. Also vermute ich einmal hundertfünfzig."	Falls Erklärung zur Schätzung durch den/die Interviewte(n) gegeben wird, in Unterkategorien gruppieren.
		1.3.2 200.000 pro Jahr	Für die Berechnung/Schätzung der Fallzahl wurde die in der Einleitung genannte Zahl von 200.000 Knie-TEPs pro Jahr in Deutschland als relevant angesehen.	„Ja weiß ich nicht, sie sagten eben was von 200.000 OPs im Jahr, ja wie viele Krankenhäuser haben wir, die das machen könnten? Vielleicht so 500, dann hätten wir so 2.000 im Durchschnitt pro Krankenhaus."	Falls Erklärung zur Schätzung durch den/die Interviewte(n) gegeben wird, in Unterkategorien gruppieren.
		1.3.3 Anzahl pro Woche/Tag	Für die Berechnung/Schätzung der Fallzahl wurde zunächst die Zahl der OPs pro Woche oder Tag geschätzt.	„Ich denke, täglich zwei drei. Und das dann malnehmen."	Falls Erklärung zur Schätzung durch den/die Interviewte(n) gegeben wird, in Unterkategorien gruppieren.
	1.4 Zahl durchschnittlich		Geschätzte Fallzahl durch Interviewte(n) in einer durchschnittlichen Klinik	„100"	Nur die Zahl kodieren
2. Klinik geringe Fallzahl	Zahl gering		Geschätzte Fallzahl durch Interviewte(n) in einem Krankenhaus mit einer geringen Anzahl an Knie TEPs	„50"	Nur die Zahl kodieren
3. Klinik hohe Fallzahl	Zahl hoch		Geschätzte Fallzahl durch Interviewte(n) in einem Krankenhaus mit einer hohe Anzahl an Knie TEPs	„1000" / „500-1000"	Nur die Zahl kodieren
	4.1 Klare Zustimmung		Interviewte*r äußert klare Zustimmung ohne Einschränkung	„Ja. Würde ich voll zustimmen."<br>„Ich glaube, dass das stimmt."	Äußerung kodieren (Satz / Absatz), ein Interview kann **nur einer Subkategorie** zugeordnet werden.<br>Begründungunter 4a kodieren
	4.2 Zustimmung mit Einschränkung		Interviewte*r äußert Zustimmung mit Einschränkung	„Da würde ich sagen teilweise, andere Faktoren haben auch einen Einfluss."<br>„Kommt darauf an. Wenn es eine hohe Zahl ist, also, wenn wirklich die Routine da ist, dann ist es sicherlich so, dass die Erfahrung letztlich zur Qualität beiträgt, zur höheren. Wenn es allerdings ohnehin, sagen wir einmal, eine spezialisierte Klinik ist, muss das nicht unbedingt sein. Also, ich denke einmal, ganz hoch muss es nicht sein, aber es sollte eine gewisse Routine da sein."	Äußerung + Begründung kodieren (Satz / Absatz), ein Interview kann **nur einer Subkategorie zugeordnet** werden. Einschränkung als Subsubkategorie kodieren. (können für die Auswertung mit 4b zusammengefasst werden)
		4.2.1 Zusammenhang komplexer	Interviewte*r vermutet, dass der Erklärungsansatz "Je mehr desto besser" komplexer ist.	„Nein, würde ich so nicht sagen. (...) Es kommt ja darauf an, auch erstmal wer es macht. (...) Also das wäre mir zu einfach."	Einschränkung kodieren (Satz / Absatz), mehrere Einschränkungen möglich. (Diese können für die Auswertung mit 4b zusammengefasst werden)
		4.2.2 Material	Interviewte*r vermutet, dass neben dem Volumen auch das Material einen Einfluss auf das Ergebnis der Operation hat.	„Das kommt erstens auf das Material an, was die Leute verwenden. Ich sage jetzt mal auf das Gelenk, auf die Qualität der Gelenke."	Einschränkung kodieren (Satz / Absatz), mehrere Einschränkungen möglich. (Diese können für die Auswertung mit 4b zusammengefasst werden)
		4.2.3 OP ohne Notwendigkeit	Interviewte*r stimmt zwar zu, dass das Ergebnis besser wird mir mehr Operationen, äußert aber die Befürchtung, dass dann viele Operationen ohne Notwendigkeit durchgeführt werden.	„Aber ich habe dann immer so die Befürchtung, dass zu viel gemacht wird, dass mehr operiert wird als überhaupt notwendig, nur um der Statistik dann die Zahlen zu liefern."	Einschränkung kodieren (Satz / Absatz), mehrere Einschränkungen möglich. (Diese können für die Auswertung mit 4b zusammengefasst werden)
		4.2.4 Kapazitätsgrenze des Krankenhauses als Obergrenze	Interviewte*r stimmt der Aussage zu, möchte allerdings eine obere Begrenzung in Berücksichtigung der Kapazitätsgrenze des Krankenhauses.	„Es sollte natürlich nicht über die Kapazitätsgrenze des Krankenhauses gehen. (...) Dann würde ich sagen, in der Tendenz ja, aber vielleicht auch mit einer Obergrenze. Vielleicht nicht unbegrenzt viele."	Einschränkung kodieren (Satz / Absatz), mehrere Einschränkungen möglich. (Diese können für die Auswertung mit 4b zusammengefasst werden)

Dimension	Subkategorie	Sub-Subkategorie	Definition	Beispiel	Kodier Regel
4. Reaktion: Je mehr desto besser		4.2.5 Routine auch negativ	Interviewte*r stimmt der Aussage zu und nimmt an, dass mit mehr Operationen die Routine zunimmt. Allerdings wird die Routine sowohl positiv als auch negativ gesehen.	„Denn ich denke mal, desto mehr Operationen ein Krankenhaus durchführt, desto größer wird die Routine mit dem Eingriff. Das würde ich auf jeden Fall sagen. Die Frage ist, wenn das zu selbstverständlich wird, also sage ich einmal, so ein bisschen die Spannung vielleicht wegfällt, weil das ist so oft, das wird gar nicht, können sich auch wieder Fehler einschleichen."	Einschränkung kodieren (Satz / Absatz), mehrere Einschränkungen möglich. (Diese können für die Auswertung mit 4b zusammengefasst werden)
		4.2.6 spezialisierte Klinik	Interviewte*r stimmt der Aussage zu, allerdings mit der Einschränkung, dass es bei spezialisierten Krankenhäusern nicht auf die Anzahl der OPs ankommt."	„Wenn es allerdings ohnehin, sagen wir einmal, eine spezialisierte Klinik ist, muss das nicht unbedingt sein. Also, ich denke einmal, ganz hoch muss es nicht sein, aber es sollt eine gewisse Routine da sein."	Einschränkung kodieren (Satz / Absatz), mehrere Einschränkungen möglich. (Diese können für die Auswertung mit 4b zusammengefasst werden)
		4.2.7 Versicherungsstatus (GKV vs. PKV)	Interviewte*r stimmt der Aussage zu, allerdings mit der Einschränkung, dass der Versicherungsstatus ebenfalls Einfluss auf das Ergebnis der Operation hat.	„[Es] ist ja nochmal ein riesen Unterschied, ob du gesetzlich krankenversichert bist oder noch privat. Also ich bin privat noch zusätzlich versichert also für Chefarzt und Einzelzimmer und sowas. Und das ist Fakt, das wissen wir alle, dass es eine Zweiklassengesellschaft ist und bei mir muss mich der Professor operieren aber ein Chefarzt hat schon seine Vorteile."	Einschränkung kodieren (Satz / Absatz), mehrere Einschränkungen möglich. (Diese können für die Auswertung mit 4b zusammengefasst werden)
		4.2.8 Beständiges Team	Interviewte*r stimmt der Aussage zu, allerdings mit der Einschränkung, dass die Erfahrung sich nur in eingespielten Teams positiv bemerkbar macht.	„Und wie gesagt, kommt darauf an, macht auch keinen Sinn, nur mal Beispiel, wenn ich das sagen darf, wenn die Operateure dauernd wechseln, weil sie woanders Angebote bekommen."	Einschränkung kodieren (Satz / Absatz), mehrere Einschränkungen möglich. (Diese können für die Auswertung mit 4b zusammengefasst werden)
		4.2.9 Eignung Ärzt*in	Interviewte*r äußert, dass die Eignung ebenfalls einen Einfluss hat.	„Es kann auch jemand sein, der noch nicht so lange Erfahrung hat, dass der einfach ein Händchen hat."	Einschränkung kodieren (Satz / Absatz), mehrere Einschränkungen möglich. (Diese können für die Auswertung mit 4b zusammengefasst werden)
		4.2.10 Anzahl Operateure/Teams	Interviewte*r äußert, dass die Anzahl der Operierenden ebenfalls einen Einfluss hat.	„Wenn wir jetzt natürlich eine Klinik mit 2.100 Operationen haben, da haben Sie mehr Operateure."	Einschränkung kodieren (Satz / Absatz), mehrere Einschränkungen möglich. (Diese können für die Auswertung mit 4b zusammengefasst werden)
	4.3 Klare Ablehnung		Interviewte*r äußert klare Ablehnung	„Nein, ich denke nicht, dass das stimmt."	Äußerung + ggf. Begründungkodieren (Satz / Absatz), ein Interview kann **nur einer Subkategorie** zugeordnet werden.
	Faktoren, die als Erklärung des Zusammenhangs zwischen Volumen und Ergebnis genannt werden einzeln kodieren.				
	4a.1 Ausstattung des Krankenhauses		Als Erklärung des Zusammenhangs zwischen Anzahl und Ergebnis wird die Ausstattung des Krankenhauses genannt: Mit mehr Fällen ist auch die Ausstattung des Krankenhauses besser.	„Wenn natürlich nur Umsatz ist, denke ich einmal auch, dass auch die nötigen Instrumente da sind, die eventuell, wenn es nur geringfügig gemacht wird in einem Krankenhaus, einfach nicht da sind. (...) Also, da sind sie einfach besser ausgestattet."	Wörter oder Sätze kodieren, die die Ausstattung des Krankenhauses als Erklärungsfaktor belegen. Mehrere Faktoren können kodiert werden.
	4a.2 Anzahl als Indikator für Zufriedenheit		Der Zusammenhangs zwischen Volumen und Ergebnis wird so erklärt, dass mehr behandelte Patient*innen in der Klinik als ein Indikator für eine bessere Klinik gesehen wird.	„Weil, da sind zehn Leute gewesen. Und das bedeutet für mich, dass es ja wahrscheinlich ein gutes Zeichen ist."	Wörter oder Sätze kodieren, die die Fallzahl als Indikator für Zufriedenheit belegen. Mehrere Faktoren können kodiert werden.

Dimension	Subkategorie	Sub-Subkategorie	Definition	Beispiel	Kodier Regel
4a. Erklärung Zusammenhang volume-outcome	4a.3 Routine/Erfahrung		Der Zusammenhangs zwischen Volumen und Ergebnis wird mit Routine (positiv), Erfahrung, Erfahrungswerten erklärt.	„Würde man erwarten, weil ja die Routine da sicher auch eine große Rolle spielt dann. Wenn also Ärzte das häufiger machen, dann kennen sie sich vermutlich besser aus als jemand, der das nur ab und zu macht." „Also je mehr Erfahrungen ein Krankenhaus damit macht desto besser werden auch die Ergebnisse."	Wörter oder Sätze kodieren, die Routine als Erklärungsfaktor belegen. Wenn Routine auch negativ gesehen wird, zusätzlich "Erfahrung/Routine negativ" kodieren. Mehrere Faktoren können kodiert werden.
		4a.3.1 Routine / Erfahrung negativ	Routine wird dabei (auch) negativ bewertet.	„Von daher würde man sagen, dass man, wenn man mehr Übung hat, besser wird. Aber es kann natürlich auch genauso gut dazu führen, dass, wenn es zu viele sind, kommt da die Routine zu stark heraus. Man wird zu routiniert und achtet nicht mehr auf jedes Detail."	Wörter oder Sätze kodieren, die Routine als negativen Einfluss belegen. Mehrere Faktoren können kodiert werden.
		4a.3.2 Spezialisierung	Interviewte*r vermutet, dass der Zusammenhang zwischen Fallzahl und Ergebnis sich durch eine steigende Spezialisierung, die sich positiv auf das Ergebnis auswirkt, erklärt.	„Also ich denke schon, dass wenn es viele Knie-OPs sind und es ist sagen wir mal die Leute sich darauf spezialisiert haben, denke ich schon, dass die Ergebnisse vermutlich im Schnitt besser sind als in anderen Krankenhaus, wo man von Hüfte über Kopf über Zehen alles machen muss, weil es einfach anfällt, ja."	Wörter oder Sätze kodieren, die die Spezialisierung als Erklärungsfaktor belegen. Mehrere Faktoren können kodiert werden.
		4a.3.3 Umgang mit Komplikationen	Interviewte*r erklärt sich den Zusammenhang zwischen Volumen und Ergebnis dadurch, dass der Umgang mit Komplikationen, falls sie auftreten, bei größerer Erfahrung besser ist.	„Und wenn mehr durchgeführt werden, gehe ich einmal davon aus, dass die Klinik auch entsprechend größer ist, wenn es jetzt, sagen wir mal, eine Durchschnittsklinik ist. Also, auf das Knie spezialisiert ist. Und von daher gibt es dann auch noch eine bessere Versorgung, falls dann irgendetwas auftreten sollte. Also, ich denke mir einmal schon, dass da größere Kliniken, ja, besser ausgestattet sind."	Wörter oder Sätze kodieren, die die Spezialisierung als Erklärungsfaktor belegen. Mehrere Faktoren können kodiert werden.
		4a.3.4 Erfahrung Ärzt*in	Interviewte*r nennt explizit die Erfahrung des/der Operateur/in oder Chirurgen/in als Erklärung des Zusammenhangs zwischen Volumen und Ergebnis.	„Ja wenn die Zahl der Operationen höher ist, dann ist das Ergebnis denke ich auch höher. Weil die Ärzte ja da ja ein bisschen versierter darin sind, in den Operationen." „Dass das Routine ist und Übung und der Arzt sich damit, wenn der so viele Operationen macht, einfach gut auskennt. Und nicht überlegen muss: „huch, wo gehört das denn jetzt hin?""	Wörter oder Sätze kodieren, die die Erfahrung des Chirurgen/der Chirurgin als Erklärungsfaktor belegen. Mehrere Faktoren können kodiert werden.
	4a.4 Eingespieltes Team		Ein gleiches, nicht wechselndes Team wird als Erklärung für den Zusammenhang zwischen Volumen und Ergebnis gesehen.	„Aber wenn das Team gleich bleibt, dann sind die eingespielt, wissen, was sie tun und dann bin ich davon überzeugt, das ist vorteilhafter, wenn die viele OPs haben."	Wörter oder Sätze kodieren, das die Zusammenspiel im Team als Erklärungsfaktor belegen. Mehrere Faktoren können kodiert werden.
4b. Sonstige Ergebnisfaktoren	Sonstige Faktoren, die das Ergebnis beeinflussen (können), die zusätzlich zum Volumen-Ergebnis-Zusammenhang genannt werden.				
	4b.1 Verhalten der Patient*innen		Interviewte*r denkt, dass auch das Verhalten der Patient*innen (nach der OP) einen Einfluss auf das Ergebnis hat.	„(...) 'Bitte rauchen Sie nicht nach der OP.' und das Erste, was die Leute machen, gehen raus vor das Krankenhaus und rauchen. Das beeinflusst natürlich auch insgesamt den Genesungsprozess und viele andere Sachen. Also das Verhalten einfach des Patienten danach. Und da kann ja der Operateur noch so gut sein."	Wörter oder Sätze, die diesen Einflussfaktor belegen, kodieren.
	4b.2 Art der Operation		Interviewte*r nimmt an, dass die Art der Operationen einen Einfluss auf das Ergebnis der Operation hat.	„Ob es jetzt sagen wir mal, ob ich sage mal normal Austauschknie ist oder was anderes."	Wörter oder Sätze, die diesen Einflussfaktor belegen, kodieren.
	4b.3 Verweildauer		Interviewte*r nimmt an, dass die Verweildauer im Krankenhaus das Ergebnis der Operation beeinflusst.	„Besser sitzen hat, vielleicht auch schneller operiert und die Liegezeit weniger ist, ich weiß es nicht. Dann verdient der auch wieder mehr Geld."	Wörter oder Sätze, die diesen Einflussfaktor belegen, kodieren.
	4b.4 Hygiene		Interviewte*r nimmt an, dass die Krankenhaushygiene das Ergebnis der Operation beeinflusst.	„Auch von der Hygiene, wenn du ein Einzelzimmer hast, gerade bei mir jetzt Protheseninfektion." „Ich würde mir hygienische Dinge vorstellen."	Wörter oder Sätze, die diesen Einflussfaktor belegen, kodieren.
	4b.5 Team		Interviewte*r meint, dass das Team Einfluss auf das Ergebnis einer Operation hat.	„Und der macht das ja auch nicht allein. Also es ist ja nicht so, dass der da allein steht. Sondern, da haben Sie ja Leute drumherum, die aufpassen."	Wörter oder Sätze, die diesen Einflussfaktor belegen, kodieren.
	5b.6 Alter		Interviewte*r meint, dass das Alter des Arztes/der Ärztin Einfluss auf das Ergebnis einer Operation hat.	„Ich meine, wenn der Operateur natürlich 63 ist und anfängt mit der Hand zu zittern, dann soll der vielleicht aufhören."	Wörter oder Sätze, die diesen Einflussfaktor belegen, kodieren.

Dimension	Subkategorie	Sub-Subkategorie	Definition	Beispiel	Kodier Regel
	4b.7 Eignung/Position der Ärzt*innen		Interviewte*r meint, dass die Eignung und/oder Position der einzelnen Ärzt*innen Einfluss auf das Ergebnis einer Operation hat.	„Da muss man auch erkennen: der ist nicht geeignet." „Der Prof. muss nicht sein, weil der operiert gar nicht mehr so viel, aber in dem Team vom Prof mit den Chefärzten und Oberärzten ist das auch das Ergebnis definitiv besser."	Wörter oder Sätze, die diesen Einflussfaktor belegen, kodieren.
	5.1 Klare Zustimmung		Interviewte*r äußert klare Zustimmung ohne Einschränkung	„Ja, da würde ich zustimmen."	Äußerung + Begründungkodieren (Satz / Absatz), ein Interview kann **nur einer Subkategorie** zugeordnet werden. Genannte Begründung als Subkategorie einfügen und ebenfalls kodieren.
		5.1.1 Gesundheit oberste Priorität	Interviewte*r beschreibt, dass er/sie weiter fahren würde für mehr Qualität, weil Gesundheit für ihn/sie oberste Priorität hat.	„Die Gesundheit steht für mich an oberster Stelle, hat höchste Priorität."	Satz/Absatz, die diese Begründung belegen, kodieren.
	5.2 Zustimmung mit Einschränkung		Interviewte*r würde für ein besseres Ergebnis eine weitere Fahrt auf sich nehmen, allerdings mit Einschränkung(en).	„Ja, also ich denke, das ist auch noch von anderen Kriterien abhängig." „Also wenn ich sage mal nicht eine Routinesache ist, würde ich definitiv immer nach einer Spezialklinik oder Spezialkrankenhaus gucken."	Äußerung + Begründung kodieren (Satz / Absatz), ein Interview kann **nur einer Subkategorie** zugeordnet werden.
		5.2.1 Besuch	Interviewte*r würde für ein besseres Ergebnis weiter fahren, gibt aber zu Bedenken, dass es für Besucher*innen dann weiter ist.	„Da ist es für den, der mich besuchen wollte, eben ein bisschen schwieriger."	Einschränkung kodieren (Satz / Absatz)
		5.2.2 Versicherungsstatus (GKV vs. PKV)	Interviewte*r äußert Bedenken, ob Patient*innen je nach Versicherungsstatus freie Krankenhauswahl haben.	„Naja, schauen Sie mal: Ich bin Kassenpatient. Und ich glaube nicht, dass eine Kasse nach meinen Wünschen geht. Die wird sich daran halten, zu sagen, hier in nächster Umgebung zu gehen, und dann war es das. Aber grundsätzlich, wenn mir die Wahl offen bliebe, dann würde ich natürlich dahin gehen, wo man davon ausgehen kann, dass es dann doch das bessere ist. Wäre also standortunabhängig."	Einschränkung kodieren (Satz / Absatz)
		5.2.3 bei komplizierten OPs	Interviewte*r würde bei komplizierten OPs eine weitere Fahrt auf sich nehmen, bei Standard-OPs jedoch (eher) nicht.	„Also wenn ich sage mal nicht eine Routinesache ist, würde ich definitiv immer nach einer Spezialklinik oder Spezialkrankenhaus gucken."	Einschränkung kodieren (Satz / Absatz)
5. Distanz Qualität		5.2.4 Verfügbarkeit von Qualitätsdaten	Interviewte*r äußert bedenken, ob er/sie Qualitätsdaten der Klinik einsehen kann.	„Na ja gut, ich weiß gar nicht, würde mich natürlich interessieren, ob man als Kunde, ob man als, Kunde sage ich schon, ob man als Patient überhaupt an so Erfolgsstatistiken kommt."	Einschränkung kodieren (Satz / Absatz)
		5.2.5 Kostenunterschied	Interviewte*r äußert Zustimmung, aber je nach Kostenunterschied zwischen den Kliniken.	„Wenn ich bei der besseren Klinik, auf einmal mehr zahlen muss. Dann würde ich mir das vielleicht überlegen."	Einschränkung kodieren (Satz / Absatz)
		5.2.6 Abhängigkeit von der Signifikanz	Interviewte*r äußert Zustimmung, aber nur bei signifikantem Ergebnisunterschied zwischen den Kliniken.	„Also, wenn da wirklich deutlich signifikant bessere Ergebnisse sind, würde ich das vermutlich machen. Wenn das nur so, sagen wir einmal, im einen steht, die Operationen gelingen zu 97 Prozent und bei dem anderen 97,3– dann würde ich es wahrscheinlich nicht machen. Also, ich müsste das Gefühl haben, das ist wirklich ein signifikant besseres Ergebnis."	Einschränkung kodieren (Satz / Absatz)
		5.2.7 Abhängigkeit von der Art des Outcomes	Interviewte*r differenziert nach der Art der Outcomes, also z. B. ob es Unterschiede in der Sterblichkeit oder in der Wiedereinweisungsrate gibt.	„Prinzipiell ja, es kommt aber vielleicht auch drauf an, in wieweit, ähm, sage ich einmal, ein erneuter Krankenhausaufenthalt ein schweres Übel wäre. Ja? Also was ich hier sehe, bei dem Beispiel, was Sie mir geschickt haben, das natürlich, was ich sagen muss, eine, ich sehe hier bei dem Beispiel eine doppelt so hohe Sterblichkeit in einem Krankenhaus, wie in dem anderen. Das wäre für mich ein Grund, das entferntere Krankenhaus definitiv zu nehmen, weil sterben möchte man auf gar keinen Fall. Da ist also eine nur eine halb so große Sterblichkeitswahrscheinlichkeit, deshalb wäre das für mich schon ein Grund."	Einschränkung kodieren (Satz / Absatz)
	5.3 Klare Ablehnung		Interviewte*r äußert klare Ablehnung	„Nein, ich möchte in der Nähe behandelt werden."	Äußerung + ggf. Begründungkodieren (Satz / Absatz), ein Interview kann **nur einer Subkategorie** zugeordnet werden.
	5.4 Frage nicht gestellt		Beim Interview wurde diese Frage nicht gestellt.		Für die Auswertung ist diese Angabe wichtig, deshalb letzten Teil der vorigen Frage und ersten Teil der nächsten Frage kodieren.
	6.1 Zahl		geschätzte Entfernung + Einheit, die Interviewte*r bereit ist zu fahren für ein besseres Ergebnis	„100 km" / „7 Stunden"	Zahl + Einheit kodieren. Falls Begründung / Einschränkung genannt wird, diese als Subkategorie mitkodieren.

Dimension	Subkategorie	Sub-Subkategorie	Definition	Beispiel	Kodier Regel
		6.1.1 Aufgrund von Erfahrungen vergangener OPs	Interviewte*r entscheidet bei Angabe der Fahrzeit aufgrund (schlechter) Erfahrung bei vergangenen Operationen.	„Also nach meiner Geschichte deutschlandweit. (…) Da wollte ich eigentlich hin, habe ich damals nicht gemacht. Also einmal war ich im anderen Krankenhaus, weil genau die Nähe wichtig war wegen meiner Tochter. (…) Aber eigentlich wollte ich in die Endoklinik und da ärgere ich mich jetzt immer noch bei der Protheseninfektion, weil da viel schief gelaufen ist. Deswegen, bei mir auf jeden Fall klar ich würde deutschlandweit fahren."	Begründung (falls angegeben) für die angegebene Fahrtzeit kodieren.
6. Distanz in km / min		6.1.2 keine Antwort	Interviewte*r kann die Entfernung nicht schätzen.	„Das kann ich jetzt nicht so-."	Anstatt der Entfernung die Antwort kodieren (für die Auswertung)
		6.1.3 je nach Operation	Interviewte*r differenziert nach der Art der Operation wie weit er/sie fahren würde.	„Aber wenn ein Spezialist beispielsweise in Hamburg ist, dann würde ich auch, und wenn es wirklich etwas Kompliziertes wäre, dann würde ich auch die tausend Kilometer fahren. Allerdings für ein neues Knie, wenn es nur um ein neues Knie geht, denke ich einmal, würde ich das sonst eher hier in der Umgebung bis 200 Kilometer machen lassen. Aber wenn es eben etwas Spezielles ist, (…) dann würde ich auch dementsprechend 600 Kilometer fahren."	Differenzierung (falls angegeben) für die unterschiedlich angegebenen Fahrtzeiten kodieren.
	6.2 Frage nicht gestellt		Beim Interview wurde diese Frage nicht gestellt.		Für die Auswertung ist diese Angabe wichtig, deshalb letzten Teil der vorigen Frage und ersten Teil der nächsten Frage kodieren.
	Faktoren, die genannt werden, die die Entscheidung für die Auswahl eines Krankenhauses zusätzlich beeinflussen. Wenn diese Faktoren im Laufe des Interviews genannt werden (nicht als Antwort auf die Frage), können sie trotzdem hier kodiert werden.				
	7.1 Persönliche Erfahrung		Persönliche Erfahrungen mit dem Krankenhausen werden als Entscheidungsfaktor genannt.	siehe Subkategorien	Wörter oder Sätze, die sich auf die persönliche Erfahren beziehen, kodieren. Die spezifischen Entscheidungsfaktoren als Subsubkategorie kodieren.
		7.1.1 eigene Erfahrung im Krankenhaus (vergangene Aufenthalte)	Persönliche Erfahrung im Krankenhaus (z. B. bei einer anderen Behandlung) wird als Entscheidungsfaktor für ein Krankenhaus genannt.	„Wenn man da schon mal war, hat das auch Einfluss."<br>„In dem Krankenhaus war ich schon, da würde ich nie hingehen"	Wörter oder Sätze, die diesen Entscheidungsfaktor belegen. Mehrere Faktoren können (einzeln) kodiert werden. In Abgrenzung zur nächsten Kategorie (7.1.2) spielen hier vergangene Aufenthalte oder vergangener Besuch von Bekannten im Krankenhaus eine Rolle.
		7.1.2 Eindruck Krankenhaus (bei Erstvorstellung)	Der aktuelle Eindruck des Krankenhaus, z. B. bei der Erstvorstellung wird als Entscheidungsfaktor für ein Krankenhaus genannt.	„Man hat ja immer eine Vorstellung oder mehrere Termine, kannst du ja machen und dann bekommst du ja auch einen Eindruck vom Krankenhaus. Das heißt ist das hektisch, ist das voll, ich sehe das auch äußerst hygienisch, da achte ich drauf als Patient."	Wörter oder Sätze, die diesen Entscheidungsfaktor belegen. Mehrere Faktoren können (einzeln) kodiert werden. In Abgrenzung zur vorigen Kategorie (7.1.1) spielen hier vergangene Aufenthalte oder vergangener Besuch von Bekannten im Krankenhaus keine Rolle, sondern der aktuelle Eindruck zählt.
	7.2 Krankenhaus		Faktoren, die sich auf das Krankenhaus beziehen werden als Entscheidungsfaktoren genannt.	siehe Subkategorien	Wörter oder Sätze, die sich auf das Krankenhaus beziehen, kodieren. Die spezifischen Entscheidungsfaktoren als Subsubkategorie kodieren.
		7.2.1 Reputation Krankenhaus	Reputation des Krankenhauses wird als Entscheidungsfaktor für ein Krankenhaus genannt.	„Ich glaube, was da für mich nur eine Rolle spielt ist wie gesagt erstmal wie gesagt der Ruf des Krankenhauses."	Wörter oder Sätze, die die Reputaion des Krankenhauses als Entscheidungsfaktor belegen. Mehrere Faktoren können (einzeln) kodiert werden. Falls nicht spezifiziert wird, ob die Reputation des Krankenhauses oder von spezifischen Ärzt*innen (7.4.5) gemeint ist, hier kodieren, da sich die Frage auf die Wahl des Krankenhauses bezieht.

Dimension	Subkategorie	Sub-Subkategorie	Definition	Beispiel	Kodier Regel
		7.2.1.1 Reputation unnötige OPs	Reputation des Krankenhauses im Hinblick auf die Praxis von wirtschaftl. vs. medizinisch notwendigen OPs wird als Entscheidungsfaktor für ein Krankenhaus genannt.	„Geht es wirklich, und deswegen anfangs auch meine Nachfrage, geht es da wirklich um gesundheitliche Interessen oder geht es da um irgendwelche ökonomischen Interessen? Weil häufig das Problem [in] Krankenhäusern ist, und das braucht man auch nicht verschweigen, dass die einfach eine bestimmte Anzahl von Operationen machen müssen, dass es sich wirtschaftlich rentiert. Das heißt, selbst, wenn kein neues Knie gebraucht wird, dann muss ein neues eingesetzt werden, weil es einfach, sagen wir einmal für das Unternehmen, oder wer auch immer, der Chef, so möchte. Und da würde ich ganz genau darauf achten. Beispielsweise in [Name eines Krankenhauses], da weiß ich ganz sicher, dass die da eine bestimmte Quote einhalten müssen, ansonsten bekommen sie Probleme. Obwohl das Knie eigentlich gar nicht operiert werden müsste. Und wenn ich so etwas höre, dann würde ich auf keinen Fall da hingehen."	Wörter oder Sätze, die diesen Entscheidungsfaktor belegen. Mehrere Faktoren können (einzeln) kodiert werden.
		7.2.2 Versorgungsumfeld des Krankenhauses	Das Versorgungsumfeld des Krankenhauses (Pflege, Personal, Nachsorge etc.) wird als Entscheidungsfaktor für die Wahl eines Krankenhauses genannt.	„Also das ganze Versorgungsumfeld auch im Krankenhaus."	Wörter oder Sätze, die diesen Entscheidungsfaktor belegen, kodieren. Mehrere Faktoren können (einzeln) kodiert werden.
		7.2.3 Zustand Krankenhaus	Der allgemeine Zustand des Krankenhauses (Modernität, Renovierung) wird als Entscheidungsfaktor genannt.	„Dass das Krankenhaus auf dem modernsten Stand ist." „Der allgemeine Zustand des Krankenhauses."	Wörter oder Sätze, die diesen Entscheidungsfaktor belegen, kodieren. Mehrere Faktoren können (einzeln) kodiert werden.
		7.2.4 Einzelzimmer	Die Verfügbarkeit eines Einzelzimmer wird als entscheidend für die Wahl eines Krankenhauses genannt.	„Also ich persönlich würde Wert auf ein Einzelzimmer legen. Und dafür würde ich auch zahlen. Und wenn das gewährleistet ist, das fließt natürlich in meine Überlegung mit ein."	Wörter oder Sätze, die diesen Entscheidungsfaktor belegen, kodieren. Mehrere Faktoren können (einzeln) kodiert werden.
		7.2.5 Trägerschaft des Krankenhauses	Die Trägerschaft des Krankenhauses (kommunal, privat) wird als Entscheidungsfaktor für die Wahl eines Krankenhauses genannt.	„Man neigt ja dazu, doch am ehesten ins städtische Krankenhaus zu gehen."	Wörter oder Sätze, die diesen Entscheidungsfaktor belegen, kodieren. Mehrere Faktoren können (einzeln) kodiert werden.
		7.2.6 Spezialisierung/ Fachlichkeit des Krankenhauses	Als Entscheidungsfaktor wird die Spezialisierung oder Fachlichkeit des Krankenhauses genannt.	„Und daher lieber Spezialklinik. Gut jetzt ist ein Kniegelenk vielleicht nicht zu speziell." „Genau, die nachgewiesene Fachlichkeit."	Wörter oder Sätze, die diesen Entscheidungsfaktor belegen, kodieren. Mehrere Faktoren können (einzeln) kodiert werden.
		7.2.7 Größe des Krankenhauses	Die Größe des Krankenhauses wird als Entscheidungsfaktor für die Wahl eines Krankenhauses genannt (Präferenz kann bei größeren oder kleineren Krankenhäusern liegen).	„Ja, eben ein stückweit auch, sagen wir einmal, die Größe. Also, eben, es sollte eine Routine da sein. Es sollte jetzt nicht so ein Eingriff sein, also, ich meine [Name eines kleines Krankenhauses] wenn Sie das jetzt schon als eines der drei Krankenhäuser haben, da ist es natürlich so, dass es eher kleiner ist. Und ich würde da ganz genau hingucken." „Jaja. Aber das ist jetzt für die Entscheidung-, weshalb ich auch nicht so für diese großen Häuser bin-."	Wörter oder Sätze, die diesen Entscheidungsfaktor belegen, kodieren. Mehrere Faktoren können (einzeln) kodiert werden.
7. Entscheidungsfaktoren		7.2.8 Kosten	Die Kosten für die OP spielen eine Rolle bei der Entscheidung für ein Krankenhaus.	„Ja, also wenn ich jetzt für eine etwas bessere Operation 5.000 Euro mehr bezahlen muss, also weil irgendwie die Krankenversicherung nur einen bestimmten Teil bezahlt, und dann kommt, dann würde ich mir natürlich sagen, und dann hängt es davon ab."	Wörter oder Sätze, die diesen Entscheidungsfaktor belegen, kodieren. Mehrere Faktoren können (einzeln) kodiert werden.
		7.2.9 Essen (wichtig / nicht wichtig)	Der/ die Interviewte geht auf die Qualität des Essens ein (wichtig / nicht wichtig für die Entscheidung).	„Das Essen (...) Wie gesagt, so, so, so Kleinigkeiten. Aber für mich sind die-, spielen solche Faktoren schon eine große Rolle." „Also auf das Essen kommt es mir nicht an, das ist mir egal. Schmeckt sowieso nie."	Wörter oder Sätze, die diesen Entscheidungsfaktor belegen, kodieren. Mehrere Faktoren können (einzeln) kodiert werden. Für die Häufigkeitsangabe nur Personen werten, die das Essen wichtig finden.
		7.2.10 WLAN	Verfügbarkeit von WLAN spielt eine Rolle bei der Wahl des Krankenhauses	„Also ich sage mal ganz pauschal WLAN und da gibt es dann doch schon große Unterschiede, heute noch."	Wörter oder Sätze, die diesen Entscheidungsfaktor belegen, kodieren. Mehrere Faktoren können (einzeln) kodiert werden.
	7.3 Empfehlungen		Faktoren, die sich auf Empfehlungen durch andere beziehen, beeinflussen die Entscheidung (Bsp. Bekannte, Ärzt*innen, Internet)	siehe Subkategorien	Wörter oder Sätze, die sich auf Empfehlungen beziehen, kodieren. Die spezifischen Entscheidungsfaktoren als Subsubkategorie kodieren.

Dimension	Subkategorie	Sub-Subkategorie	Definition	Beispiel	Kodier Regel
		7.3.1 Internetrecherche	Informationen aus dem Internet (Erfahrungsberichte, Homepage der Klinik etc.) werden als Entscheidungsfaktor für ein Krankenhaus genannt.	„Letztlich ist das für uns wahrscheinlich eher so, dass man irgendwie im Internet nach Erfahrungsberichten sucht. Und dann einmal guckt, was die Leute da so schreiben (…)" „Ansonsten würde ich sagen, ich würde wirklich mir die Homepage der Klinik angucken, würde mir angucken, was es vielleicht für Berichte zu dem Arzt oder so gibt."	Wörter oder Sätze, die diesen Entscheidungsfaktor belegen, kodieren. Mehrere Faktoren können (einzeln) kodiert werden.
		7.3.2 Empfehlung (Haus)ärzt(in)	Die Empfehlung des (Haus)Arztes / der (Haus)Ärztin wird als Entscheidungsfaktor für ein Krankenhaus genannt.	„Wobei ich da mich auf meinen Arzt verlassen habe. Aber da muss man auch sagen, vielleicht ist das noch ein Kriterium, Tipp des Arztes."	Wörter oder Sätze, die diesen Entscheidungsfaktor belegen, kodieren. Mehrere Faktoren können (einzeln) kodiert werden.
		7.3.3 Empfehlung Bekannte/Familie	Für die Entscheidung für ein Krankenhaus werden Empfehlungen durch Bekannte/Familie herangezogen.	„Ich höre mich im Bekanntenkreis um, wer da schon mal war"	Wörter oder Sätze, die diesen Entscheidungsfaktor belegen, kodieren. Mehrere Faktoren können (einzeln) kodiert werden.
	7.4 Personal		Faktoren, die das Personal betreffen, werden als Entscheidungsfaktoren genannt (Ärzt*innen, Pflege)	siehe Subkategorien	Wörter oder Sätze, die sich auf das Personal beziehen, kodieren. Die spezifischen Entscheidungsfaktoren als Subsubkategorie kodieren.
		7.4.1 Welcher Arzt / Ärztin operiert mich?	Interviewte*r finden es entscheidend zu wissen welcher Arzt / welche Ärztin die OP durchführen wird, z. B. um sich ein Bild zu machen und Vertrauen aufzubauen.	„(…) und dann auch wer operiert mich. Ich will dann auch vorher wissen, wie ist das Team, [ich] informiere mich immer vorher, also ich gehe da nicht ins kalte Wasser und vertraue nur den Ärzten."	Wörter oder Sätze, die diesen Entscheidungsfaktor belegen, kodieren. Mehrere Faktoren können (einzeln) kodiert werden. In Abgrenzung zu 7.4.3 und 7.4.5 liegt hier der Fokus auf WER, d.h. der/die Interviewte möchten explizit wissen, WER sie operiert und dass diese Entscheidung festgelegt ist.
		7.4.2 Aufklärung	Für den/ die Interviewte ist es wichtig eine gute Aufklärung über die Operation und mögliche Folgen zu erhalten.	„Und der hat mir alles erklärt, wie die Herzoperation funktioniert, wie das geht, wirklich. Der hat sich eine Mühe gegeben. Und das war angenehm. Hat mir alles aufgezeichnet. Was man machen kann. Und dann auch nochmal erklärt, dass wir eine Vene entnehmen aus dem linken Bein: ‚Von da bis da, werden vier Schnitte. Die ziehen wir raus. Und das regeneriert sich dann.' Das war-. Und damit habe ich jetzt, seitdem ich aus dem Krankenhaus bin, Probleme. Nicht mit dem Herzen, das ist wunderbar geworden. Also da kann ich immer nur gratulieren. Die Kollegen haben gut gearbeitet. Und die haben wahrscheinlich auch die Vene gut rausgenommen. Nur, einem zu sagen: ‚Ja, damit haben Sie danach Schwierigkeiten. Und da müssen wir mal drauf aufpassen.', das haben sie nicht."	Wörter oder Sätze, die diesen Entscheidungsfaktor belegen. Mehrere Faktoren können (einzeln) kodiert werden.
		7.4.3 Vertrauen zum Operateur / zur Operateurin	Interviewte*r erklärt, dass das Vertrauen zum Operateur/ zur Operateurin ein Entscheindungsfaktor für die Wahl eines Krankenhauses ist.	„Vertrauen zum Operateur."	Wörter oder Sätze, die diesen Entscheidungsfaktor belegen. Mehrere Faktoren können (einzeln) kodiert werden. In Abgrenzung zu 7.4.1 und 7.4.5 liegt hier der Fokus auf Vertrauen, d. h. Interviewte sollten explizit das Stichwort "Vertrauen" äußern, damit diese Kategorie kodiert wird.
		7.4.4 Freundlichkeit Personal	Die Freundlichkeit des Personals oder Sympathie zu den behandelnden Ärzt*innen wird als Entscheidungsfaktor für ein Krankenhaus genannt.	„Also, wie gesagt, Freundlichkeit vom Personal, (…) so Kleinigkeiten. Aber für mich sind die-, spielen solche Faktoren schon eine große Rolle." „Der Kollege (…), hat aber einen Kölschen Dialekt gesprochen, wir haben uns wunderbar verstanden. War wirklich toll. Wenn Sie dann Kölsch hören. Und dann fragt man direkt: ‚Mann, Sie kommen doch auch-?' ‚Ja.' sagt der Herr, ‚Ich war sechs Jahre-, studiert.' Hat ihm wunderbar gefallen in Köln. Er war jetzt in Berlin schon mehrere Jahre, hat aber den Dialekt aus Köln nicht abgelegt. >lacht< Finde ich schön."	Wörter oder Sätze, die diesen Entscheidungsfaktor belegen. Mehrere Faktoren können (einzeln) kodiert werden.

Dimension	Subkategorie	Sub-Subkategorie	Definition	Beispiel	Kodier Regel
		7.4.5 Reputation Ärzt*in	Reputation der Ärzt*in wird als Entscheidungsfaktor für ein Krankenhaus genannt.	„Hier der Orthopäde, der hier operiert, [soll einen] sehr guten Ruf haben." „Weil ich da zu so einem berühmten Professor gegangen bin."	Wörter oder Sätze, die diesen Entscheidungsfaktor belegen. Mehrere Faktoren können (einzeln) kodiert werden. In Abgrenzung zu 7.2.1 muss hier explizit die Ärzt*in angesprochen werden. In Abgenzung zu 7.4.1 und 7.4.3 ist hier das Stichwort "Ruf" / "Reputation" wichtig und nicht so sehr "wer" oder das "Vertrauen".
8. Verständnisfragen Ergebnisse Volume-Outcome	8.1 Allgemein		Interviewte*r hat generell Fragen zu der Darstellung der Ergebnisse des Reviews, angewandt auf Krankenhäuser in der Umgebung.	„Das verstehe ich nicht. Also d. h. es sind nicht die richtigen Zahlen."	Wörter/Frage kodieren, die belegen, dass der/die Interviewte Verständnisprobleme mit der Darstellung der Ergebnisse hat. Einzelne Fragen/ Probleme als Subkategorie kodieren
	8.2 Gründe für Unterschiede		Interviewte*r fragt nach den Gründen für die Unterschiede zwischen verschiedenen Krankenhäusern.	„Eine Frage, was mich da mal interessieren würde, ich weiß nicht, ob Sie mir das sagen können, wenn die Frage kommt, weiß man denn, warum ein Krankenhaus besser abschneidet als das andere? Kann man das ermitteln?"	Wörter/Frage kodieren, die diese Verständnisfrage belegen.
	8.3 Studienhintergrund		Interviewte*r hat Fragen, die sich auf die eingeschlossenen Studien beziehen (Bsp. eingeschlossene Patienten).	„Aber hier aber dann nochmal eine Frage, jetzt du jetzt, die alles durchgeguckt hat, was ist da die Hauptaltersgruppe gewesen? Weißt du, ist ja wichtig ob 65 oder auch frühere oder ob das jetzt 65-80 ist."	Wörter/Frage kodieren, die diese Verständnisfrage belegen.
	8.4 Lokalisation Krankenhäuser		Interviewte*r hat Fragen zur Lokalisation der dargestellten Krankenhäuser, z. B. weil es mehrere Krankenhäuser mit gleichem Namen in der Umgebung gibt.	„Ja, aber Sankt Josef ist in, ist das in [Name eines Ortes] oder in [Name eines Ortes]?"	Wörter/Frage kodieren, die diese Verständnisfrage belegen.
	8.5 Outcomes		Interviewte*r hat Fragen zu den vorgestellten Outcomes, Bsp. Sterblichkeit (nicht gewusst, dass dies eine Komplikation der Knie-TEP OP ist).	„Das hätte ich jetzt nicht gedacht, dass man dann, was das Knie betrifft, dass man wegen-, da. Selbst wenn es da Komplikationen gibt, dass man davon sterben kann. Ja, also gut."	Wörter/Frage kodieren, die diese Verständnisfrage belegen.
	8.6 Männchen Rundung		Interviewte*r versteht die Darstellung in Männchen nicht oder hat Fragen zu der Rundung der Prozentzahlen.	„Aber wie, wie kann-, wie kann das denn sein, das fällt mir hier gerade mal auf, bei dem [Krankenhaus1] und [Krankenhaus2]. Da sind ja jetzt von den-. Ist ja jetzt überhaupt niemand angemarkert. Also, die sind ja alle grün."	Wörter/Frage kodieren, die diese Verständnisfrage belegen.
	8.7 Fiktive Zahlen		Interviewte*r versteht die Einschränkung der Interviewerin nicht, dass es sich um berechnete Zahlen aus einer Formel handelt, nicht um die tatsächlichen Zahlen des dargestellten Krankenhauses. Er/sie fragt sich, ob es sich dann um fiktive Zahlen handelt.	„Das verstehe ich nicht. Also d. h. es sind nicht die richtigen Zahlen." „(...) Aber ich verstehe, dass da so eine Art Prognose ist, die aufgrund der Erfahrungen aus anderen Kliniken im Durchschnitt eben passen müsste."	Wörter/Frage kodieren, die diese Verständnisfrage belegen
8. Wichtigkeit Fallzahl	8.1 Zustimmung		Interviewte*r äußert Zustimmung auf die Frage, ob anhand der Zahlen ein wichtiger Einfluss erkennbar ist.	„Ja, das würde ich schon sagen."	Äußerung + Begründungkodieren (Satz / Absatz), ein Interview kann nur einer Subkategorie zugeordnet werden.
	8.2 Keine klare Aussage		Aussage kann weder als Zustimmung noch als Ablehnung gewertet werden.	„Hmm, das weiß ich jetzt nicht, ob das wichtig ist. Ein Zusammenhang ist da, aber ist der wichtig?"	Äußerung + Begründung kodieren (Satz / Absatz), ein Interview kann nur einer Subkategorie zugeordnet werden.
	8.3 Ablehnung		Interviewte*r äußert Ablehnung auf die Frage, ob anhand der Zahlen ein wichtiger Einfluss erkennbar ist.	„Sicherlich, also, wenn ich sehe, dass das erste einen achtfachen Wert hat, ist das erst einmal sehr viel bei [Klinik1] gegenüber [Klinik2]. Praktisch ist natürlich 0,8 Prozent immer noch nicht sehr viel. (...) Also, da würde ich sagen, die Werte unterscheiden sich zwar objektiv, aber für mich wäre das jetzt kein Kriterium, unbedingt nach [Klinik3] zu gehen, im Vergleich zu [Klinik2] oder so." „Also wenn ich mir so die Zahlen ansehe, so bis auf die ersten beiden Ergebnisse, ja, hat das kaum Einfluss."	Äußerung + ggf. Begründungkodieren (Satz / Absatz), ein Interview kann nur einer Subkategorie zugeordnet werden.
	9.2 Zustimmung		Interviewte*r würde mind. ein Krankenhaus ausschließen aufgrund der Ergebnisse.	„Ja, in das [Krankenhaus mit geringster Anzahl] würde ich jetzt nicht mehr gehen."	Äußerung + Begründungkodieren (Satz / Absatz), ein Interview kann nur einer Subkategorie zugeordnet werden.

Dimension	Subkategorie	Sub-Subkategorie	Definition	Beispiel	Kodier Regel
9. Ausschluss Krankenhäuser	9.3 Keine klare Aussage		Interviewte ist sich nicht sicher, ob er/sie mind. ein Krankenhaus ausschließen würde oder Interviewte*r würde ein Krankenhaus ausschließen, aber aus anderen Gründen als den Ergebnissen aus dem Review.	„Die Daten regen zum Nachdenken an, aber ob ich da eins ausschließen würde, weiß ich nicht."<br>„In das Krankenhaus [Name] würde ich nicht gehen, aber nicht aufgrund Ihrer Ergebnisse."	Äußerung + Begründungkodieren (Satz / Absatz), ein Interview kann nur einer Subkategorie zugeordnet werden.
	9.4 Ablehnung		Interviewte*r würde nach Besprechung der Ergebnisse weiter in alle Krankenhäuser gehen (z. B. weil der Unterschied als nicht groß gesehen wird oder andere Faktoren eine größere Rolle spielen als die dargestellten Ergebnisse).	„Nein, ich würde trotzdem in alle Krankenhäuser gehen."	Äußerung + Begründungkodieren (Satz / Absatz), ein Interview kann nur einer Subkategorie zugeordnet werden.
10. Meinung Mindestmenge hoch	10.1 Positiv		Interviewte*r findet es positiv oder ok, wenn weniger Krankenhäuser Knie-TEPs anbieten.	„Ja das fände ich gut, da würde ich sowieso nicht hingehen."<br>„An sich wäre das gut, oder? Also, fände ich."	Äußerung + Begründungkodieren (Satz / Absatz), ein Interview kann nur einer Subkategorie zugeordnet werden.
		10.1.1 Mindestmenge pro Ärzt*in	Interviewte*r stellt sich zusätzlich eine Mindestmenge pro Ärzt*in vor, um ausreichend Erfahrung sicherzustellen.	„Ja wäre ich sogar froh, (…) aber was nützt [es] mir, wenn die 70 noch von drei oder zehn verschiedenen Ärzten gemacht werden, dann hat ja jeder Arzt nur 5 oder 7. Dann geht das ja gar nicht. Ja also ich beziehe das sogar auf den Arzt, auf das Team, auf die Operateure, weil was bringt mir das wenn ich jetzt 200 habe und auf 10 Ärzte verteile, das ist auch zu wenig. Also ich spreche ja auch vom Arzt, dass ein Arzt mindestens 100 haben muss."	Satz/ Absatz, der als Beleg für die Forderung nach einer Mindestmenge pro Ärzt*in dienen, kodieren.
		10.1.2 Mehr Spezialisierung	Nach Ansicht der/des Interviewten würde es durch Hochsetzung der Mindestmenge eine Spezialisierung der Krankenhäuser geben. Das wird positiv gewertet.	„Ich bin sowieso dafür, dass sich die Krankenhäuser spezialisieren, dass man zum Beispiel irgendwo nur Herzoperationen macht und bei dem anderen nur diese Kniesachen und Hüftsachen, wissen Sie? Dass also nicht jeder machen kann, was er will. Da wäre ich sehr dafür!"	Satz/ Absatz, der als Beleg für die positive Betrachtung von mehr Spezialisierung dienen, kodieren.
	10.2. Positiv mit Einschränkung		Tendenziell wird eine Hochsetzung der Mindestmenge von der/dem Interviewten/m positiv gesehen, allerding mit Einschränkungen, z. B. für andere Personen.	„Für mich wäre es ok, aber für andere, die nicht so mobil sind, ist es nicht gut."	Äußerung + Begründung kodieren (Satz / Absatz), ein Interview kann nur einer Subkategorie zugeordnet werden.
		10.2.1 Höher als 50, aber unter der Fallzahl des kleinsten KH	Interviewte*r sieht eine Hochsetzung der Mindestmenge positiv, findet allerding eine Hochsetzung auf z. B. 300, sodass das kleinste dargestellte Krankenhaus wegfallen würde, zu hoch. (z. B. bei Interviewteilnehmer*innen, die sehr nah an großen Krankenhäusern wohnen.)	„Also ich fände es schon gut, wenn die hochgesetzt würde. Ob es nun 300 sein muss, weiß ich nicht. [Ich würde] [Name eines Krankenhauses] ja nicht ausschließen. Dann können es ja 250 sein?. >Beide lachen.< Also 50 finde ich eigentlich schon-, finde ich schon wenig. Das [ist] ja dann pro Woche eine OP. Das finde ich sehr wenig."	Satz/ Absatz, der als Beleg für diese Einschränkung dient, kodieren.
		10.2.3 Ländliche Versorgung	Interviewte*r sieht eine Hochsetzung der Mindestmenge positiv unter der Voraussetzung, dass die Versorgung in ländlichen Regionen weiterhin sichergestellt ist.	„Für mich wäre das noch okay, also zurzeit. (…) Wenn ich auf dem Land wohne, aber dann habe ich ja eh das Problem, wo ist das nächste Krankenhaus? Das ist ja eh alles dann problematischer."	Satz/ Absatz, der als Beleg für diese Einschränkung dient, kodieren.
	10.3 Keine klare Äußerung		Interviewte findet es zwar ok, wenn weniger Krankenhäuser die OP anbieten können, aber kann auch Nachteile erkennen. Die Aussage hat keine Tendenz.	„Das weiß ich jetzt nicht, ob ich das gut finde."	Äußerung + Begründung kodieren (Satz / Absatz), ein Interview kann nur einer Subkategorie zugeordnet werden.
		10.3.1 Besser: Ergebnisse in allen Krankenhäusern verbessern.	Interviewte findet hat keine Tendenz zur Hochsetzung der Mindestmenge und bevorzugt stattdessen die Suche nach den Gründen der Unterschiede und folglich die Verbesserung aller Kliniken.	„Ja, deswegen wäre so mein Ansatz, man müsste halt eben rausfinden, warum eine Klinik schlechter abschneidet als die andere? Und dann müsste man eben die Ursachen zu bekämpfen. Weil es hätte wahrscheinlich jeder Patient Interesse daran, nah an seinem Wohnungsort, wenn möglich, den Eingriff durchziehen zu können."	Satz/ Absatz, der als Beleg für diesen Ansatz dient, kodieren.
		10.3.2 Markt regelt von selbst	Interviewte*r denkt, dass es keine Regularien bracht, denn der Markt würde, wenn die Ergebnisse bzw. Fallzahlen bekannt sind, es selbst regeln, d. h. Patient*innen würden automatisch in Krankenhäuser mit höheren Fallzahlen oder besseren Ergebnissen gehen.	„Und jetzt könnte man natürlich sagen, wenn man die Zahlen jetzt veröffentlicht, also auch Zahlen, wie viele Operationen insgesamt ein Krankenhaus durchführt, dann würde das vielleicht schon der Markt erledigen."	Satz/ Absatz kodieren, der als Beleg dient, dass der Markt die Steuerung von Patienten selbst regeln wird.

Dimension	Subkategorie	Sub-Subkategorie	Definition	Beispiel	Kodier Regel
		10.3.3 Kapazitätsgrenzen	Interviewte*r äußert Bedenken, dass eine Hochsetzung der Mindestmenge dazu führt, dass die Kapazitätsgrenze der Krankenhäuser, die dann noch Knie-TEPs anbieten, ggf. ausgereizt wird.	„Das hängt dann auch von der Kapazität der anderen Kliniken ab."	Satz/ Absatz kodieren, der als Beleg dient, dass es Befürchtungen bzgl. der Kapazitätsgrenze gibt.
	10.4 Negativ		Interviewte*r findet es schlecht, wenn weniger Krankenhäuser diese OP anbieten.	„Nein, das fände ich nicht gut, dann würden evtl. Krankenhäuser geschlossen."	Äußerung + Begründung (als neue Subkategorie) kodieren (Satz / Absatz), ein Interview kann nur einer Subkategorie zugeordnet werden.
		10.4.1 OPs pro Operateur*in	Interviewte*r sieht eine Hochsetzung der Mindestmenge kritisch, mit der Begründung, dass aus dieser Zahl nicht hervorgeht, wie viele Operationen ein*e Ärzt*in durchführt.	„Das sind dann auch mehrere, so dass sie-. Das sind zwar mehr Operationen, ja. Aber der Einzelne, wieviel der macht, das geht daraus ja nicht hervor."	Satz/ Absatz kodieren, der als Beleg für diese Begründung dient.
		10.4.2 Schließung Krankenhäuser negativ	Interviewte*r befürchtet, dass durch Hochsetzen der Mindestmenge Krankenhäuser schließen müssten und lehnt dies ab.	„Also wenn man jetzt aus heutiger Sicht die Corona-Krise noch sieht, dann würde ich sagen, man sollte die Fallzahl nicht erhöhen. Weil, wenn es nur noch nach Fallzahlen geht und nicht mehr, dass da auch eine medizinische Versorgung bleibt, dann schließt man ja irgendwann immer mehr Kliniken. Und das fände ich nicht sinnvoll."	Satz/ Absatz kodieren, der als Beleg für diese Begründung dient.
		10.4.3 Mobilität älterer Menschen	Interviewte*r sieht eine Hochsetzung der Mindestmenge kritisch mit der Begründung, dass die Mobilität älterer Menschen eingeschränkt ist.	„Also spontan würde ich sagen, dass es nicht sinnvoll ist. (…) Also mich jetzt vielleicht nicht so, aber ich denke schon, dass vielleicht auch ältere Menschen, die dann nicht mehr so mobil sind, dass das für die dann schon auch entscheidend sein kann."	Satz/ Absatz kodieren, der als Beleg für diese Begründung dient.
		10.4.3 Ergebnisunterschied zu gering	Interviewte*r sieht die Unterschiede, die aus der Berechnung anhand des Reviews folgen, als zu gering an, um daraus Maßnahmen abzuleiten.	„Wissenschaftlich muss man das, meine ich, um so etwas zu machen, auf jeden Fall stärker durchleuchten. Weil mir die Angaben hier definitiv nicht ausreichen."	Satz/ Absatz kodieren, der als Beleg für diese Begründung dient.
		10.4.5 Operationen ohne Notwendigkeit	Interviewte*r befürchtet, dass durch Hochsetzen der Mindestmenge eine Ausweitung der Indikationsstellung stattfindet wird und mehr "unnötige" OPs durchgeführt werden.	„Und wenn das jetzt heißt, ja wenn sie keine 50 Knieoperationen machen, keine 150, dürfen Sie gar nicht mehr operieren, dann ist ja die Frage, wie kriegen Sie die denn zusammen? (…) Praktisch muss man sagen, wenn jetzt so eine Klinik A gesagt bekommt, ja Sie müssen aber 150 Operationen machen, was passiert denn dann? Dann fangen die an, Ärzte zu belabern, dass sie einmal mehr krankschreiben sollen oder mehr versuchen, die aus anderen Einzugsbereichen Leute zu sich zu gewinnen. Und machen Werbung und solche Sachen. Das ist ein bisschen schwierig. Für mich persönlich hätte das, glaube ich, bei dem Bereich, keine konkreten Auswirkungen. Sage ich jetzt so."	Satz/ Absatz kodieren, der als Beleg dient, dass es Befürchtungen gibt, dass ein Hochsetzen der Mindestmenge dazu führt, dass die Indikationsstellung für Knie-TEPs ausgeweitet wird.
		10.4.6 Ländliche Versorgung	Interviewte*r sieht eine Hochsetzung der Mindestmenge negativ weil befürchtet wird, dass die Versorgung in ländlichen Regionen nicht sichergestellt ist.	„Also ich würde sagen, nein, fände ich nicht gut, weil (…) das würde ja möglicherweise bedeuten können, dass ländliche Kliniken, die einfach nicht so ein Einzugsgebiet haben, so auch für ältere Leute, die auch dahin müssten"	Satz/ Absatz kodieren, der als Beleg für diese Begründung dient.

Regeln:

Bei Subsubkategorien auch Subkategorie mitkodieren.
Fragliche Fälle in eigenen Code "mit Nadja besprechen" + Memo wohin Tendenz
Generell eher ganze Sätze als einzelne Wörter kodieren, wenn der Zusammenhang für die Nutzung als Zitat relevant ist.
