# Supplementary material for: Perspective of potential patients on the hospital volume-outcome relationship and the minimum volume threshold for total knee arthroplasty: a qualitative focus group and interview study
Source: BMC Health Serv Res. 2021 Jul 2;21:633. doi: 10.1186/s12913-021-06641-8 (PMC8249216; doi:10.1186/s12913-021-06641-8)
Supplement: Supplementary file 5 — Additional file 5. Selected quotes translation. [file 12913_2021_6641_MOESM5_ESM.pdf]

Additional file 5: Selected quotes translation

Perspective of potential patients on the hospital volume-outcome relationship and the minimum volume threshold for total knee arthroplasty: A qualitative focus group and interview study

Authors:

M.Sc. Charlotte M. Kugler charlotte.kugler@uni-wh.de (1), PhD Karina K. De Santis desantis@leibniz-bips.de (1), MPH Tanja Rombey tanja.rombey@uni-wh.de (1), PhD Kaethe Goossen kaethe.goossen@uni-wh.de (1), M.Sc. Jessica Breuing jessica.breuing@uni-wh.de (1), M.Sc. Nadja Koensgen nadja.koensgen@uni-wh.de (1), Dr. Tim Mathes tim.mathes@uni-wh.de (1), Simone Hess simone.hess@uni-wh.de (1), Dr. René Burchard rene.burchard@uni-wh.de (2, 3, 4), Dr. Dawid Pieper dawid.pieper@uni-wh.de (1)

(1) Institute for Research in Operative Medicine, Witten/Herdecke University, Ostmerheimer Str. 200, 51109 Cologne, Germany

(2) Department of Trauma Surgery and Orthopaedics, Lahn-Dill-Kliniken, Rotebergstr. 2, 35683 Dillenburg, Germany

(3) Department of Health, Witten/Herdecke University, Alfred-Herrhausen-Straße 50, 58448 Witten, Germany

(4) School of Medicine, Univerity of Marburg, Baldingerstraße, 35032 Marburg, Germany

Corresponding author: Charlotte M Kugler, charlotte.kugler@uni-wh.de, Tel: +49 221 9895742

Question	Category	German	English
		**Focus group**	
4	High volume	„Ich habe das nur mal überlegt, dass ja drei Ärzte operieren im ganzen Krankenhaus. Und die an einem Tag jeder	'I was just thinking this through, with three doctors operating in the whole hospital. And they would do two

Question	Category	German	English
		zwei OPs machen würden. Und wenn ich dann mal 300 Tage, sage ich mal, rechne...“ (B4)	surgeries a day each. And then, when I calculate 300 days, let's say...’ (B4)
5	Yes	„Je mehr die machen, umso besser wird es. Je mehr Routine haben die doch. (...) Also, wie bei der Schraube an Autos. Je mehr Kupplungen der in verschiedene Modelle einbaut umso besser ist er. Umso schneller geht es.“ (B1)	‘The more they do, the better it will get. The more routine they have, after all. (…) Well, like with screws in cars. The more clutches a guy builds into different models, the better he is. The faster it gets.’ (B1)
5	Yes, but…	„Es kann natürlich auch das Gegenteil sein, die werden mit der Zeit oberflächlicher. (...) Je mehr sie machen. Aber ich will das nicht hoffen und auch nicht unterstellen.“ (B4)	‘Of course, it can also be the opposite; they become more superficial over time. (…) The more they do. But I don't want to hope so and I don't want to imply that either.’ (B4)
5	Yes, but…	„Aber es gibt ja immer Fälle wo es nicht funktioniert. (…) Die Frage ist auch immer, ob da der Operateur was dafür kann. Ist das jetzt jemandes Schuld oder wäre das sowieso passiert?“ (B4)	‘But there are always cases where it doesn't work. (...) The question is also whether the surgeon can do anything about it. Is it someone's fault or would it have happened anyway?’ (B4)
9	Yes	„Ja ist doch logisch“ (B1)	‘Yes, obviously’ (B1)
9	Yes, but…	„Es sei denn, es ist wirklich mit Nachuntersuchungen im Operationsort verbunden.“ (B2)	‘Unless it really involves follow-up visits to the operating hospital.’ (B2)
9	Yes, but…	„Ich denke auch, es kommt auf das Alter und den Zustand des Patienten an. (…) Mit dem Arzt reden. Das können auch viele ältere Menschen nicht alleine, weil sie es nicht verstehen. Wenn sie das Hörgerät nicht an haben, weil sie verwirrt sind oder was auch immer.“ (B3)	‘I also think it depends on the age and the condition of the patient. (...) Talk to the doctor. Many older people can't do that alone because they don't understand it. When they don't have their hearing aid on, because they are confused, or whatever.’ (B3)
10	Distance: short	„Das ist schwer. Also so zwei, drei Stunden, mehr würde ich nicht sagen. Nach der OP muss ich eh abgeholt werden. Ich kann nicht selber fahren.“ (B3)	‘That's hard. So, maybe two, three hours, I wouldn't say any longer. After surgery I have to be picked up anyway. I can't drive myself.’ (B3)
10	Distance: quality-dependent	„Vielleicht gibt es in Deutschland ein Krankenhaus, das dafür steht, dass es exzellente Arbeit in diesem Bereich macht.“ (B2)	‘Maybe there is a hospital in Germany that is renowned for doing excellent work in this field.’ (B2)
**Interviews**			
1	Average	„Wenn man 200 Tage arbeitet, so viel an einem Tag dann. Oder zwei an einem Tag könnten es schon sein. Ach, dann sage ich mal 400.“ (B20)	‘If one works 200 days, that's as many in one day, then. Or it could even be two a day. Oh, I'd say 400, then.’ (B20)
4	Yes	„Würde man erwarten, weil ja die Routine da sicher auch eine große Rolle spielt dann. Wenn also Ärzte das häufiger machen, dann kennen sie sich vermutlich besser aus als jemand, der das nur ab und zu macht.” (B8)	‘One would expect that, because routine surely plays a big role as well. So if doctors do it more often, then they probably know about it better than someone who only does it once in a while.’ (B8)
4	Yes, but..	„Das würde ich mal sagen, zum Teil richtig. Denn (…) desto mehr Operationen ein Krankenhaus durchführt, desto größer	‘I would say so, partially correct. Because (…) the more surgeries a hospital performs, the more routine

Question	Category	German	English
		wird die Routine mit dem Eingriff. (…) wenn das zu selbstverständlich wird, (…) können sich auch wieder Fehler einschleichen." (B14)	there is with the procedure. (…) when it gets too routine, (…) mistakes may also slip in again.' (B14)
4	Yes, but…	„Also es liegt ja nicht nur am Krankenhaus, sondern es liegt ja auch an dem Operateur und alles, was da drum herum ist." (B19)	'Well, it's not just the hospital, but also the surgeon and everything else that goes with it.' (B19)
5	Yes	„Ja auf jeden Fall. (…) die Gesundheit hat für mich höchste Priorität." (B21)	'Yes, definitely. (...) health has the highest priority for me.' (B21)
5	Yes, but…	„Also wenn [es] (…) nicht eine Routinesache ist, würde ich definitiv immer nach einer Spezialklinik (…) gucken." (B12)	'So if [it] (…) is not a routine thing, I would definitely always look for a specialised clinic (…).' (B12)
7	Hospital-related	„Na ja, also, definitiv ein stückweit auch der Ruf. Also irgendwie Mundpropaganda, beziehungsweise, ja, Image." (B10)	'Yeah, well, definitely the reputation to some extent. Word of mouth, somehow, or rather, yes, image.' (B10)
7	Recommendation	„Manchmal hört ja auch schon mal auf das, was Bekannte oder Familienangehörige sagen oder im Kollegenkreis spricht man auch mal darüber (…)." (B8)	'Sometimes one listens to what your acquaintances or family members say or talks about it among colleagues (…).' (B8)
7	Staff-related	„Am besten wäre natürlich, wenn ich jemand kennen würde, der auch da gewesen ist und sagt, da wirst du auch gut gepflegt, da wirst du gut versorgt, da sind die Ärzte nett." (B15)	'Naturally, it would be best if I knew someone who has also been there and who would say, you will be well cared for there, you will be well looked after there, the doctors are nice.' (B15)
7	Personal experience	„Also, da ich damals im [Name eines Krankenhauses] gelegen habe (…), würde ich da gerne wieder hingehen dann. Man hat da seine Erfahrungen gesammelt (…). Ich würde das bevorzugen." (B19)	'Well, because back then I stayed at the [hospital name] (…), I would like to go there again, then. One has made experiences there (…). I would prefer that.' (B19)
8	Agreement	„Ich halte das für erheblich. Also 4,2 zu 5,1 sind ja immerhin fast 20 Prozent." (B17)	'I consider that substantial. After all, 4.2 to 5.1 is almost 20 percent.' (B17)
8	No clear statement	„Naja er ist vorhanden, aber ob er jetzt wichtig ist, also so weit ging jetzt die Zahl nicht auseinander." (B13)	'Well, it exists, but whether it is important, well, the numbers didn't differ that much.' (B13)
8	Disagreement	„Also wenn ich mir so die Zahlen ansehe, so bis auf die ersten beiden Ergebnisse, ja, hat das kaum Einfluss. (…) Aber ist eigentlich nur die erste Zahl. Aber 0,9 zu 0,2, ja, ist auch nicht so gravierend." (B16)	'So, when I look at the numbers, well, apart from the first two results, yes, that hardly has any influence. (…) But it's actually only the first number. But 0.9 to 0.2, even that is not so serious.' (B16)
9	Agreement	„Ja hundert Prozent, ja das ist ja viel zu wenig. (...) Dafür ist es zu komplex." (B7)	'Yes, one hundred percent, yes, that is far too low. (…) It's too complex for that.' (B7)
9	No clear statement	„Ich hätte noch eine leichte Tendenz hin zu dem [3.] Krankenhaus. (…) Aber wie gesagt, ich würde jetzt nicht sagen: ‚Um Himmels Willen, nicht nach [Name des 1. Krankenhauses].' Also soweit geht es jetzt mit Sicherheit nicht." (B14)	'I still have a slight tendency towards the [3rd] hospital. (...) But as I said, I would not say: "For heaven's sake, not to [1st hospital name]." So this is definitely not as far as it goes.' (B14)
9	Disagreement	„Also ausschließen glaube ich würde ich nichts. Nochmal, da kommt es auf den Operateur an." (B12)	'Well, I guess I wouldn't rule out anything. Again, it depends on the surgeon.' (B12)

Question	Category	German	English
10	Positive	„Ich bin sowieso dafür, dass sich die Krankenhäuser spezialisieren, dass man zum Beispiel irgendwo nur Herzoperationen macht und bei dem anderen nur diese Kniesachen und Hüftsachen, wissen Sie? Dass also nicht jeder machen kann, was er will." (B18)	'Anyway, I'm in favour of hospitals specialising so that, for example, one does just the heart operations at one location and only those knee and hip things at another one, you know? So that not everyone can do what they want.' (B18)
10	Positive with restrictions	„Also wenn ich das Ergebnis hier sehe, (...), im Schnitt würde ich dann sagen, es wäre besser, wenn das hochgesetzt wird. (…) Es gibt ja Menschen, (…) für die die Erreichbarkeit dann noch wichtiger ist." (B15)	'So, looking at the result here, (…), on average I would say it would be better if that were raised. (...) There are people (...) for whom accessibility is even more important.' (B15)
10	Negative	„Nein, ich fänd[e] das insgesamt nicht gut. (…) ich persönlich warne davor, dass da (…) ein Aspekt des Profits reinkommt. (...) manchmal habe ich den Eindruck, dass einige OPs, das muss jetzt nicht unbedingt Knie sein, dass die halt gemacht werden, damit halt die Quote erfüllt wird und [das] müsste vielleicht nicht sein. (…) Alternativen (..) werden aber gar nicht so unbedingt gesagt, weil einfach Zugzwang der Ärzte oder des Krankenhauses ist, weil einfach die Zahlen nicht stimmen." (B12)	'No, overall, I don't find that good. (…) I personally warn against that there is (…) a profit aspect to it. (…) sometimes I get the impression that some surgeries, not necessarily the knee, that they are just done to meet the quota and might not be necessary. (…) Alternatives (…) are not necessarily even mentioned because doctors or hospitals are simply under pressure because the numbers are simply not right.' (B12)

Note. Symbols in brackets show participant identification number
